# Injectable Peptide‐Based Functional Amyloid Hydrogels as Tunable Depots for Sustained Drug Delivery

**DOI:** 10.1002/advs.78568

**Published:** 2026-10-29

**Authors:** Ranjit Shaw, Niyamat M. A. Chimthanawala, Shouvik Manna, Sadhana Sathaye, Samir K. Maji

**Affiliations:** ^1^ Department of Biosciences and Bioengineering Indian Institute of Technology Bombay Mumbai India; ^2^ Department of Pharmaceutical Sciences and Technology Institute of Chemical Technology Mumbai India; ^3^ Sunita Sanghi Center of Aging and Neurodegenerative Diseases (SCAN) Indian Institute of Technology Bombay Mumbai India

**Keywords:** diabetes, functional amyloid hydrogel, insulin delivery, peptide self‐assembly, sodium alginate, sustained drug delivery

## Abstract

Sustained delivery of therapeutic proteins remains a major challenge because rapid degradation and diffusion necessitate frequent dosing, compromising therapeutic efficacy. Here, we present an injectable peptide‐based functional amyloid hydrogel as a versatile drug depot that preserves protein stability while enabling sustained cargo release. As a representative therapeutic application, insulin delivery for diabetes was employed to validate the platform. It exhibited excellent biocompatibility and injectability, providing controlled insulin release over 90 h while preserving its structural stability. Following a single subcutaneous administration in diabetic rats, it restored normoglycemia within 30 min and maintained it for 60 h, followed by a rebound at 66 h, representing a much longer period of glucose maintenance than free insulin. Long‐term in vivo biosafety studies confirmed the absence of systemic toxicity following repeated administration. To demonstrate the tunability of this platform, it was further engineered with sodium alginate as a representative bioadditive, thereby enabling tunable release kinetics and extending sustained insulin release while maintaining injectability and biocompatibility. Collectively, these findings establish peptide‐based functional amyloid hydrogels, either alone or engineered with a bioadditive, as a safe, injectable, and tunable platform for sustained therapeutic delivery, with diabetes serving as a representative proof of concept for broad biomedical applications.

## Graphical abstract

An injectable and tunable peptide‐based functional amyloid hydrogel provides sustained insulin release while preserving insulin stability and bioactivity. The hydrogel enables prolonged glycemic regulation in vivo, exhibits excellent biocompatibility, and shows no detectable IAPP and α‐Syn heterologous cross‐seeding, highlighting its potential as a safe platform for long‐acting therapeutic protein delivery.

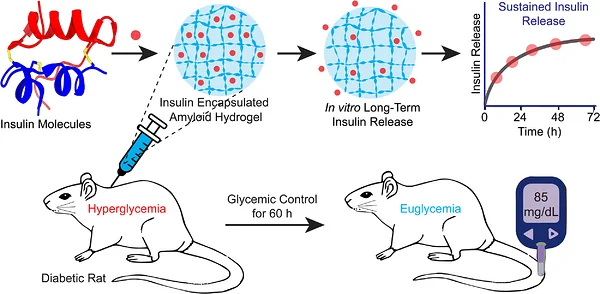

## 1 Introduction

Therapeutic proteins and peptides have transformed the treatment of numerous chronic diseases owing to their remarkable specificity and potent biological activity [1]. Nevertheless, their clinical efficacy is frequently limited by rapid diffusion from the site of administration, structural destabilization, enzymatic degradation, and short in vivo residence times, necessitating repeated dosing to maintain therapeutic concentrations [1, 2, 3]. Frequent administration not only compromises patient compliance but also increases treatment costs, injection‐site complications, and fluctuations in drug exposure, thereby reducing overall therapeutic efficacy [2]. Consequently, there is a pressing need for injectable biomaterial depots capable of protecting therapeutic cargos while enabling sustained, predictable release over extended periods.

Insulin therapy represents a clinically important example of this broader challenge. Diabetes mellitus (DM) is marked by chronic hyperglycemia, arising from inadequate insulin secretion, reduced insulin sensitivity, or both [4, 5, 6]. Thus, hyperglycaemic states occur when endogenous insulin secretion or cellular sensitivity is compromised in patients with DM [6]. Globally, DM affects over 540 million individuals and is expected to increase to 783 million by 2045, making it one of the fastest‐growing non‐communicable diseases worldwide [7, 8]. In such a scenario, deaths from DM account for over 11% of all‐cause mortality worldwide [7]. Furthermore, chronic hyperglycemia predisposes patients to severe, diabetes‐associated micro‐ and macrovascular complications brought on by a persistently high blood glucose level (BGL), such as cardiovascular disease, nephropathy, retinopathy, neuropathy, and stroke, substantially increasing morbidity and mortality [9].

Exogenous insulin administration remains the cornerstone therapy for Type 1 diabetes and advanced Type 2 diabetes. However, repeated insulin administration is associated with poor compliance, injection‐site inflammation, tissue damage from frequent dosing, adipose tissue accumulation at the injection site that causes uneven insulin absorption, and an increased risk of glucose‐level variability, which can lead to hypoglycemia [1, 10, 11, 12, 13]. Moreover, its therapeutic benefit is limited by rapid diffusion and degradation at the injection site after subcutaneous administration [10, 13], thereby necessitating multiple daily injections to maintain glycemic control. Consequently, insulin serves as an ideal representative therapeutic protein for evaluating sustained long‐acting drug delivery platforms that can improve therapeutic efficacy while reducing dosing frequency, which is highly desirable for improving patients’ quality of life.

Three‐dimensional hydrated networks, known as hydrogels, have emerged as attractive biomaterial platforms for sustained drug delivery due to their advantageous characteristics, including high water content, interconnected porous architecture, biocompatibility, injectability, and biodegradability, which collectively facilitate cargo encapsulation, nutrient transport, and controlled molecular diffusion [1, 14, 15, 16]. Their physicochemical properties can be readily tailored to regulate drug‐release kinetics, making them attractive candidates for localized, sustained delivery of therapeutic proteins and peptides [14, 16]. A plethora of injectable hydrogels for long‐term sustained delivery of insulin and other anti‐diabetic therapeutics has emerged to reduce injection frequency, facilitate patient compliance, and minimize burst release. Recent developments include thermoresponsive, glucose‐responsive, pH‐responsive, and nature‐inspired hydrogel systems. The first major class comprises thermoresponsive hydrogels, which undergo sol–gel transition at physiological temperature to form injectable in situ depots for sustained therapeutic release [17, 18, 19, 20]. A second class includes pH‐responsive hydrogels for the sustained release of insulin by employing a polymer network that swells in the neutral environment of the small intestine or blood, enabling on‐demand insulin release due to increased pH compared with the acidic pH in the stomach [17, 21]. Another widely investigated class is glucose‐responsive hydrogels for the prolonged delivery of insulin in response to changes in BGLs, thereby mimicking physiological conditions and creating a closed‐loop insulin delivery system [22, 23, 24, 25, 26, 27]. Finally, naturally derived and supramolecular hydrogels have been reported to be biocompatible injectable matrices that provide sustained release via molecular interactions and network‐mediated diffusion [19, 20, 28]. Despite these advancements, most of these systems face several challenges, including burst drug release, long‐term biodegradation, biocompatibility issues, clearance from the body, batch‐to‐batch variability, complex manufacturing, and scalability, which collectively hinder their widespread clinical translation [1, 2, 14, 16]. Therefore, there remains a critical need for injectable biomaterial platforms that simultaneously provide drug cargo stability and sustained, predictable release.

Functional amyloids have recently emerged as a unique class of supramolecular biomaterials capable of addressing these challenges [29, 30, 31]. Amyloids are supramolecular protein/peptide assemblies composed of cross‐β‐sheet‐rich structures that exhibit exceptional mechanical robustness and remarkable resilience to harsh physicochemical environments, including proteolytic degradation [32, 33, 34, 35, 36, 37, 38]. Importantly, recent studies have fundamentally reshaped the perception of amyloids by demonstrating that naturally occurring food‐derived amyloid fibrils are safe nutritional ingredients, exhibiting excellent in vitro and in vivo biocompatibility with no detectable toxicity, thereby establishing functional amyloids as a safe and versatile class of biomaterials [30, 34, 39]. In parallel, functional amyloids have emerged as promising long‐acting drug depots that can stabilize therapeutic proteins, protect them from premature degradation, and enable sustained release while preserving their biological activity, thereby making them an attractive biomaterial platform for therapeutic protein delivery [29, 40, 41]. Consequently, functional amyloid hydrogels have found increased applications in drug delivery [16, 29, 34, 40], tumor spheroid modeling [42, 43], matrices for stem cell differentiation [44, 45, 46, 47, 48], wound healing [49], and other biotechnological applications [50, 51, 52, 53]. Despite these advances, the potential of peptide‐based functional amyloid hydrogels as a modular platform for sustained therapeutic delivery remains unexplored. Addressing this challenge could establish functional amyloid hydrogels as a new class of tunable drug depots applicable to a broad range of biologics across a wide range of therapeutic conditions.

Building upon these advances, we hypothesized that a peptide‐inspired functional amyloid hydrogel could serve as a universal long‐acting injectable depot for sustained therapeutic delivery by simultaneously stabilizing encapsulated biologics and enabling controlled release.

To validate this concept, insulin was selected as a representative protein therapeutic and Type 1 diabetes as a representative disease model, owing to the stringent requirements for preserving insulin stability and bioactivity during prolonged release. Thus, amyloid‐peptide‐based hydrogels were fabricated via self‐assembly in response to pH and comprehensively characterized through a combination of biophysical analyses to establish their structural, mechanical, and physicochemical properties. The hydrogels exhibited high drug encapsulation efficiency (>90%), thixotropic self‐healing, and excellent injectability, biocompatibility, and sustained insulin release for ∼72 h, while preserving the structural integrity and functional activity of the released protein. Furthermore, its therapeutic efficacy was validated in Streptozotocin (STZ)‐induced Type 1 diabetic rats, in which a single subcutaneous administration maintained normoglycemia for ∼60 h (2.5 days), followed by a comprehensive long‐term biosafety evaluation that revealed no detectable systemic toxicity, together with the absence of detectable amylin/islet amyloid polypeptide (IAPP) and α‐Synuclein (α‐Syn) cross‐seeding activity, supporting the safety of the amyloid biomaterial as an injectable depot.

Recognizing that different therapeutic cargos require distinct release profiles, we further hypothesized that the release characteristics of this peptide‐based functional amyloid depot could be rationally tuned without disrupting its fibrillar architecture. To demonstrate this modular design strategy, sodium alginate (S.A.) was incorporated as a representative biocompatible bioadditive to engineer composite hydrogels with tunable diffusion properties [54]. The formulation exhibited optimal structural characteristics and enabled more efficient insulin encapsulation, with an extended sustained release of ∼90 h while preserving insulin structural integrity and functionality. Collectively, these findings establish peptide‐based functional amyloid hydrogels, either alone or engineered with suitable bioadditives, as a safe and tunable platform for sustained therapeutic delivery, with insulin serving as a representative cargo and diabetes as a proof‐of‐concept disease model for broader biomedical applications.

## 2 Results

### 2.1 Fabrication and Biophysical Characterization of Peptide‐Based Amyloid Hydrogels

The peptide‐based functional amyloid hydrogel system employed in the current work builds upon our previous studies, in which its self‐assembly, pH‐stimulated gelation, self‐healing properties, and rheological characterization were established. These amyloid‐based hydrogels have subsequently been explored for diverse biotechnological applications, including in vitro and in vivo stem‐cell differentiation [45, 46], where their ability to provide sustained growth‐factor release was also demonstrated [47]. Further, this class of hydrogel has been used for tumor spheroid modeling using both cells and xenograft‐derived tissues [42], wound healing in normal and diabetic rats [49], and cell‐adhesion matrices without integrin involvement [44, 53, 55]. However, their potential to function as a sustained therapeutic drug depot has not been previously explored. In the present study, we therefore investigated the ability of this functional amyloid hydrogel to serve as a subcutaneous inulin depot and further demonstrated that it can provide tunable release through bioadditive incorporation, preserve the structural integrity and in vivo glucose‐lowering activity of the encapsulated insulin during prolonged storage and release, and achieve sustained glycemic control in a diabetic rat model.

To achieve this, two peptide‐based functional amyloid hydrogels were fabricated via a pH‐dependent method and comprehensively characterized as established before [42, 49]. We selected two functional amyloid (FA) hydrogels, denoted FA1 and FA2, for their excellent biocompatibility and thixotropicity (Figure S1). After peptide synthesis (Figures S2 and S3), hydrogels were reconstituted in buffers (Figure 1A) according to the established protocol [42, 43], and formation was confirmed by the traditional gel inversion test (Figure 1B). The morphology and constituent network were confirmed using transmission electron microscopy (TEM), scanning electron microscopy (SEM), and atomic force microscopy (AFM), which revealed that highly ordered nanofibrils constitute the hydrogel network (Figure 1C–E; Figure S4), as reported before [42, 49], with an overall porous architecture (∼5–10 µm pore size distribution) (Figure 1F,G). As expected, both the hydrogels exhibited viscoelastic behavior, as indicated by the frequency sweep experiment (Figure 1H) with a higher storage modulus (G’) compared to the loss modulus (G″). Further, the hydrogel's thixotropicity is established by a step‐strain rheology study (Figure 1I), consistent with our previously reported results [45, 46, 47, 56].

**FIGURE 1:**
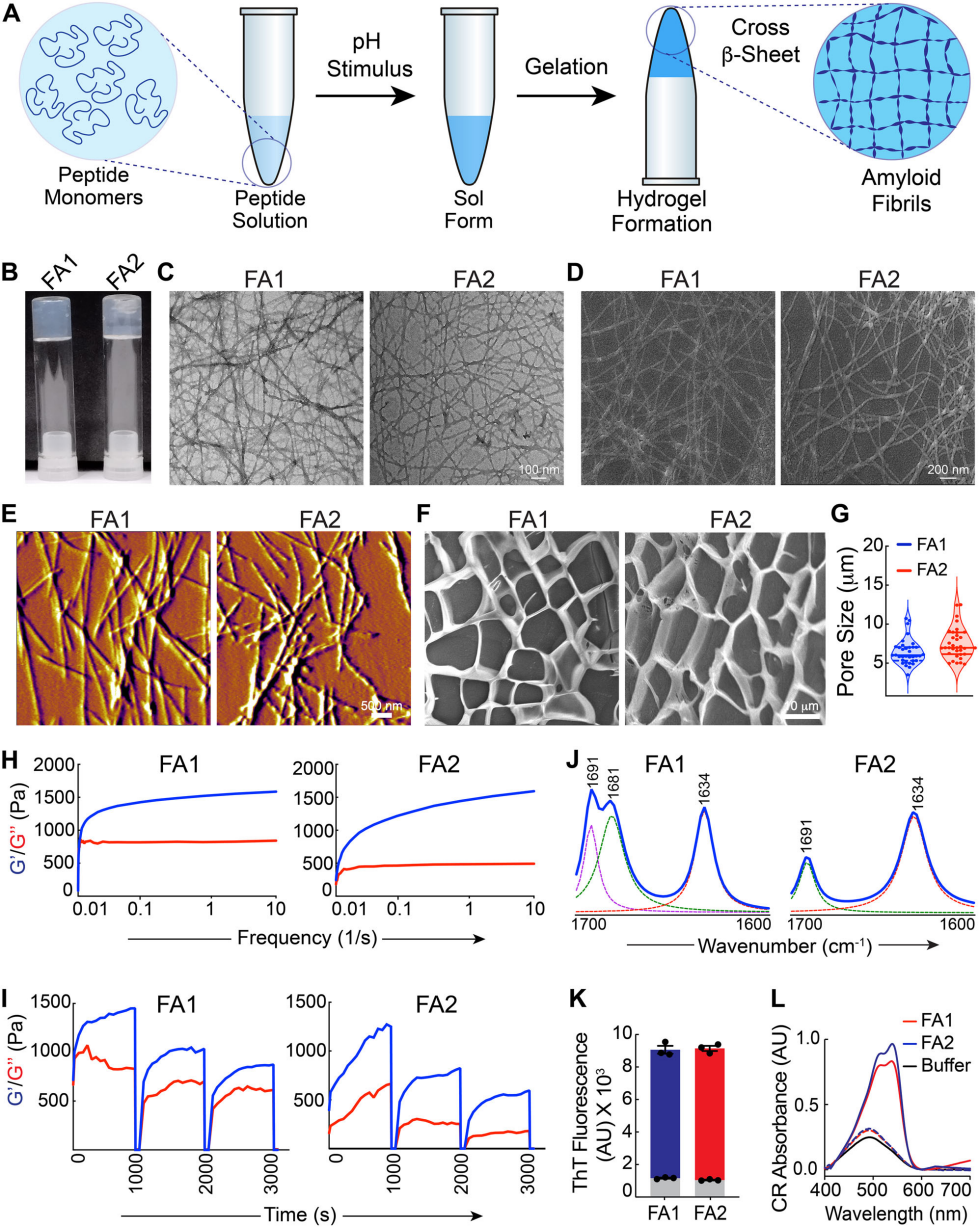
Biophysical characterization of the peptide‐based functional amyloid hydrogels. (A) Schematic illustration of the pH‐triggered self‐assembly process used to fabricate the peptide‐based functional amyloid hydrogels. (B) Representative gel inversion test confirming the successful formation of these hydrogels. (C) Transmission electron micrographs of dried hydrogel demonstrating typical long, unbranched amyloid fibrils. The scale bar is 100 nm. (D) Scanning electron micrographs showing fibrillar surface morphology of the dried hydrogels. The scale bar is 200 nm. (E) Atomic force micrographs of the hydrogels, depicting their nanofibrillar architecture, forming an interconnected mesh‐like framework. The scale bar is 500 nm. (F) Cryo‐scanning electron micrographs of both hydrogels, demonstrating their 3D porous architecture. The scale bar is 2 µm. (G) Violin plots showing the pore size distribution of hydrogels calculated from Cryo‐SEM images. (H) Frequency sweep rheological test at 37°C, showing G′ and G″ as a function of oscillation frequency, demonstrating the viscoelastic nature of the hydrogels (G′ > G″). (I) Step‐strain rheological analysis at 37°C demonstrating reversible gel‐sol transitions and rapid recovery of the storage modulus following repeated high‐ and low‐strain cycles, indicative of thixotropic self‐healing behavior of the hydrogels. (J) FTIR spectra of hydrogels exhibiting the presence of peaks of typical amide I bands corresponding to cross‐β‐sheet‐rich amyloid structures. (K) ThT fluorescence assay, demonstrating amyloid formation by the hydrogels through enhanced fluorescence emission at 480 nm. The corresponding peptide monomers are shown as controls (grey bars inside each individual bar). (L) CR binding assay demonstrating a characteristic increase in absorption accompanied by a bathochromic (red) shift in the absorption maximum, implying the hydrogel's amyloid‐like feature. —— denote the corresponding peptide monomers as controls. Data were plotted and analyzed using GraphPad Prism v9.5.1 and ImageJ v1.54 (NIH, Bethesda, USA), as applicable, and figures were prepared and assembled using Adobe Illustrator 2023 v27.0.

These properties play a pivotal role in drug encapsulation and the formulation's injectability [14, 16]. Further, in the present formulation conditions, the hydrogel constituting fibrils showed cross‐β‐sheet structural signature in Fourier transform infrared (FTIR) spectroscopy (Figure 1J), bind both amyloid‐specific dyes, Thioflavin T (ThT by increased fluorescence at 480 nm; Figure 1K), and Congo Red (CR by UV–vis spectroscopy; Figure 1L), indicating that the fibrillar network responsible for hydrogel formation exhibited characteristic amyloid‐like features.

### 2.2 Hydrogel Swelling and Biocompatibility with Functional Characterization of Insulin‐Loaded Amyloid Hydrogels

Because hydrogel swelling determines both drug‐release kinetics and tissue compatibility, careful control of it is crucial for sustained therapeutic efficacy [14, 16, 57]. Moreover, it is important to evaluate whether the release of the encapsulated payload is due to simple diffusion or to hydrogel swelling, which accelerates release kinetics. Thus, a hydrogel swelling assay was performed, which revealed that the swelling ratios of both hydrogels were ∼10% after 24 h (Figure 2A). Therefore, these amyloid hydrogels do not expand significantly in volume. A primary concern regarding amyloid‐based biomaterials is their potential cellular toxicity, as amyloid aggregates are implicated in numerous human diseases, including Alzheimer's and Parkinson's diseases [58, 59, 60, 61]. Therefore, to evaluate the suitability of these hydrogels for drug delivery applications, their cytocompatibility was assessed using the MTT assay of NIH 3T3 (fibroblast) cells. The hydrogels exhibited more than 90% viable cells after 24 h of incubation (Figure 2B), and bright‐field imaging of the cells grown on amyloid hydrogels revealed normal cell morphology (Figure 2C and Figure S5A), suggesting that these amyloid hydrogels are biocompatible. Further, hemocompatibility was also assessed using the hemolysis assay, which exhibited ∼2% hemolysis, equivalent to that of the negative control [i.e., phosphate‐buffered saline (PBS)], indicating our hydrogel's biocompatibility with blood (Figure 2D; Figure S5B,C).

**FIGURE 2:**
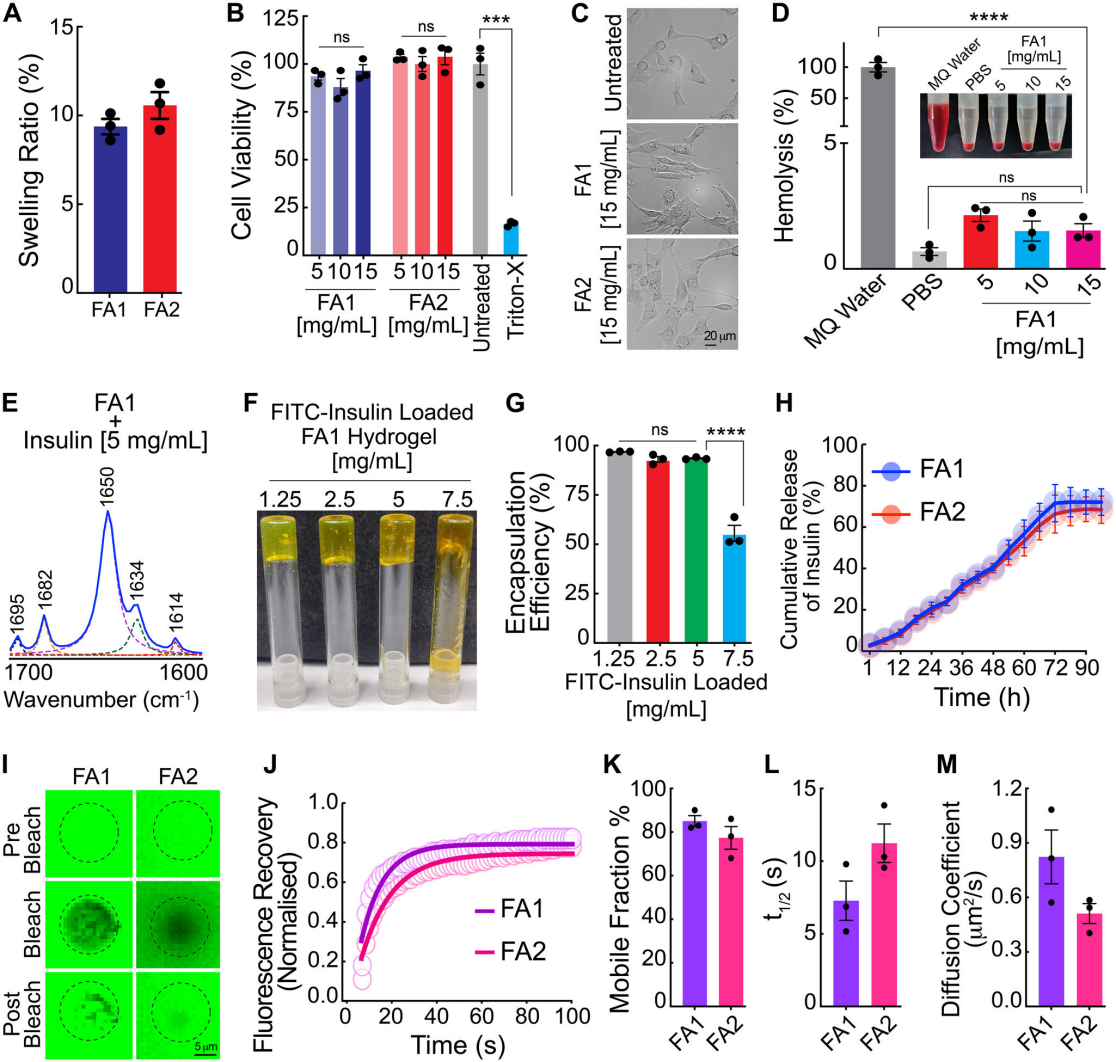
Swelling behavior, biocompatibility, and insulin release characteristics of the peptide‐based functional amyloid hydrogels. (A) Swelling ratio of the hydrogels over time, demonstrating minimal volumetric expansion under physiological conditions. (B) Cytocompatibility of the FA1 hydrogel evaluated by MTT assay using NIH 3T3 fibroblasts cultured on hydrogels prepared at peptide concentrations of 5 mg/mL, 10 mg/mL, and 15 mg/mL for 24 h. (C) Representative bright‐field micrographs of NIH 3T3 cells cultured on hydrogel‐coated well plates, showing normal cell morphology and cell adhesion. (D) Hemocompatibility assessment of the FA1 hydrogel by hemolysis assay. Representative photographs of the assay solutions (inset) and the corresponding % hemolysis quantification are shown. Intact erythrocyte pellets were observed beneath the clear supernatant of the PBS and FA1 hydrogel groups, whereas complete hemolysis in the MQ‐treated positive control produced a bright red supernatant without a visible pellet. (E) FTIR spectra of FITC‐insulin‐encapsulated FA1 hydrogel, exhibiting the characteristic amide I band corresponding to the alpha‐helical structure, confirming successful encapsulation while preserving its native protein conformation. (F) Representative images of inverted tubes containing FA1 hydrogels encapsulating increasing concentrations of FITC‐insulin, demonstrating successful hydrogel formation following insulin loading. (G) Encapsulation efficiency of the FA1 amyloid hydrogel in loading different concentrations of FITC‐insulin. (H) In vitro cumulative release assay of insulin from both hydrogels over 90 h, demonstrating sustained protein release. (I) Representative fluorescence images of the FRAP study using FITC‐insulin‐loaded hydrogels, depicting pre‐bleach, bleach (0 s), and post‐bleach phases. (J) Normalized fluorescence recovery curves of FITC‐insulin encapsulated inside them, showing high recovery. This illustrates molecular diffusion of the loaded cargo within the hydrogel networks. (K–M) Quantitative analysis of the FRAP experiments showing the % mobile fraction (K), fluorescence recovery half‐time (L), and calculated diffusion coefficients (M) of FITC‐insulin during the FRAP assay. For B, D, and G, the data represent the mean ± S.E.M. (*n* = 3), and the statistical significance was determined using a one‐way ANOVA, followed by a Tukey's (B and D) and Dunnett's (G) multiple‐comparison test [^****^ *p* < 0.0001, ^***^ *p* < 0.0002, and ^ns^ *p* > 0.1234 with a 95% confidence interval]. Data were plotted and analyzed using GraphPad Prism v9.5.1, OriginPro 2021 v9.8.0, and ImageJ v1.54 (NIH, Bethesda, USA), as applicable, and figures were prepared and assembled using Adobe Illustrator 2023 v27.0.

To use these amyloid hydrogels as a possible drug‐delivery depot to treat diabetes, FITC‐insulin was encapsulated within them, as confirmed by the presence of an alpha‐helical secondary conformation in FTIR spectra (Figure 2E; Figure S6A) and fluorescence microscopy of the FITC‐insulin‐loaded hydrogels (Figure S6B). This encapsulation didn't alter either the hydrogel architecture or the secondary structure of the amyloid networks, as confirmed by the existence of cross‐β‐sheet in FTIR spectroscopy. The encapsulation efficiency (EE) of the payload inside the amyloid hydrogels was more than 95% up to a concentration of 5 mg/mL FITC‐insulin (Figure 2F,G; Figure S6C,D). However, it was unable to efficiently encapsulate FITC‐insulin at concentrations above 5 mg/mL, as evidenced by the inverted‐tube images (Figure 2F; Figure S6C).

To evaluate sustained release of the encapsulated payload from the amyloid hydrogels, the in vitro release assay was performed with a 5 mg/mL FITC‐insulin‐loaded hydrogel. Both amyloid hydrogels exhibited sustained, controlled release for up to 72 h, as shown in Figure 2H. The cumulative release profiles were further fitted to zero‐order, first‐order, Higuchi [62], and Korsmeyer‐Peppas kinetic models [63] (Table S1). Among these, the zero‐order model showed the highest coefficient of determination (R^2^ = 0.9569 for FA1 and 0.9568 for FA2), indicating that insulin is released at a nearly constant rate over time. This behavior suggests that the amyloid hydrogel network serves as an effective reservoir, enabling sustained and controlled insulin release.

To examine whether the diffusivity of the encapsulated cargo is influenced by its interactions with the amyloid fibrillar network, we performed fluorescence recovery after photobleaching (FRAP) experiments on FITC‐insulin‐loaded hydrogels, which exhibited high fluorescence recovery and a mobile fraction (∼80%) (Figure 2I–K). The recovery half‐time (Figure 2L) is short, while the diffusion coefficient (Figure 2M) is relatively high, indicating that the amyloid hydrogels effectively encapsulated insulin while permitting its diffusion, thereby enabling sustained release.

### 2.3 Stability of Encapsulated Insulin in the Hydrogel Delivery System during Storage and Release

For therapeutic application, the hydrogel formulation must preserve the stability of encapsulated insulin during long‐term refrigerated storage. To examine this, insulin‐loaded hydrogels were stored at 4°C for up to 6 months to assess their shelf‐life by comprehensively evaluating the conformational, chemical, and biological integrity of the released insulin using circular dichroism (CD) spectroscopy, matrix‐assisted laser desorption/ionization‐time‐of‐flight mass spectrometry (MALDI‐TOF MS), ion‐pair high‐performance liquid chromatography (IP‐HPLC), tris‐tricine sodium dodecyl sulfate‐polyacrylamide gel electrophoresis (SDS‐PAGE) under non‐reducing conditions, and in vivo functional efficacy studies in Type 1 diabetic rats (Figure 3A). IP‐HPLC, MALDI‐TOF MS, and Tris‐tricine SDS‐PAGE analyses revealed identical chromatographic profiles, retention times, molecular masses, and electrophoretic band patterns (as evidenced by a single band corresponding to its molecular weight) for hydrogel‐released insulin compared with freshly dissolved insulin. Further, no detectable degradation products were observed after 6 months of storage, confirming the preservation of its chemical integrity, primary structure, and molecular integrity (Figure 3B–D; Figure S7A–C). Additionally, CD spectroscopy showed that the hydrogel‐released insulin retained the characteristic α‐helical secondary structure (two negative minima at ∼208 and ∼222 nm) of native insulin (Figure 3E; Figure S7D), with no detectable conformational changes after 6 months of storage, confirming its structural stability. Lastly, subcutaneous administration of hydrogel‐released insulin to STZ‐induced diabetic rats elicited a robust glucose‐lowering response comparable to that of fresh insulin (Figure 3F; Figure S7E), supporting the retention of its in vivo biological activity due to the maintenance of chemical/structural integrity following long‐term storage (up to 6 months at 4°C).

**FIGURE 3:**
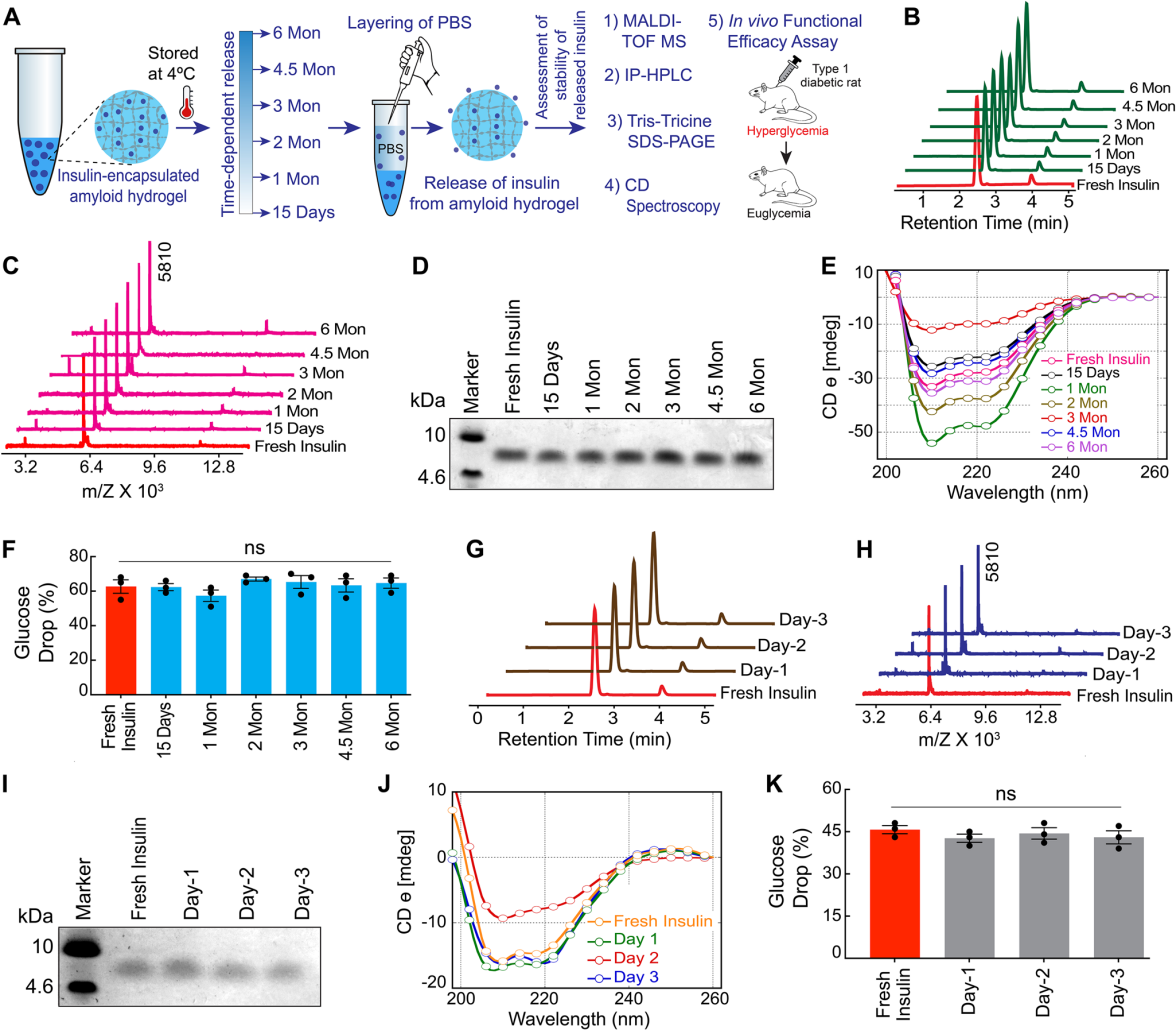
Structural and functional stability of insulin encapsulated within the FA1 peptide‐based functional amyloid hydrogel during long‐term storage at 4°C and sustained release at 37°C. (A) Schematic illustration of the experimental workflow used to evaluate the structural integrity and biological activity of insulin following long‐term storage within the amyloid hydrogel at 4°C. (B‐F) Stability assessment of insulin encapsulated within the FA1 hydrogel during storage at 4°C for up to 6 months. (B) IP‐HPLC chromatograms showing no detectable degradation peaks. (C) MALDI‐TOF mass spectra confirming preservation of the molecular mass of insulin. (D) Tris‐Tricine SDS‐PAGE under non‐reducing conditions demonstrating retention of insulin integrity. (E) Far‐UV CD spectra showing preservation of the native α‐helical secondary structure of insulin following storage. (F) In vivo functional efficacy assay in STZ‐induced Type 1 diabetic rats demonstrating that insulin released after long‐term storage retains its glucose‐lowering activity. (G‐K) Stability assessment of insulin released from the FA1 hydrogel during sustained release at 37°C for up to 3 days. (G) IP‐HPLC chromatograms. (H) MALDI‐TOF mass spectra. (I) Tris‐Tricine SDS‐PAGE under non‐reducing conditions. (J) Far‐UV CD spectra. (K) In vivo functional efficacy assay of insulin released from FA1 hydrogel, verifying the conformational and functional integrity of insulin maintained throughout the release period of 3 days. Collectively, these analyses demonstrate that the amyloid hydrogel preserves the primary and secondary structure and the biological function of encapsulated insulin during both prolonged storage and physiological release. For F and K, the data represent the mean ± S.E.M. (*n* = 3), and the statistical significance was determined using a one‐way ANOVA, followed by a Dunnett's multiple‐comparison test [^ns^ *p* > 0.1234 with a 95% confidence interval]. Data were plotted and analyzed using GraphPad Prism v9.5.1, OriginPro 2021 v9.8.0, KaleidaGraph v4.03, and ImageJ v1.54 (NIH, Bethesda, USA), as applicable, and figures were prepared and assembled using Adobe Illustrator 2023 v27.0.

Although long‐term refrigerated storage establishes the formulation's shelf life, it is equally important to verify that insulin remains structurally and functionally intact during sustained release under physiological temperature. Therefore, we next evaluated the insulin stability during release from the FA1 amyloid hydrogel at 37°C to confirm that prolonged residence within the hydrogel does not induce degradation or loss of bioactivity during therapeutic release. The various biophysical studies confirmed that insulin released from the amyloid hydrogel remained chemically and structurally intact (no peaks/signals/bands of breakdown products) throughout the three‐day release period, and in vivo glucose‐lowering studies in diabetic rats verified its chemical/structural integrity under physiological conditions (Figure 3G–K).

### 2.4 In Vivo Hypoglycaemic Effect of Insulin‐Encapsulated Amyloid Hydrogel in Type 1 Diabetic Rats

Based on the sustained in vitro release of insulin, we tested the efficacy of the selected representative hydrogel, FA1, in preclinical models to treat hyperglycemia. We first created a Type 1 diabetic rat model by injecting STZ intraperitoneally (i.p.) into male Wistar rats and then administered the FA1+insulin formulation (5 mg/mL), insulin alone (5 mg/mL), PBS (10 mm, pH 7.4), FA1 alone, and insulin glargine (Lantus: 12IU/animal), a clinically established long‐acting insulin analog, as the positive control (Figure 4A). We observed no blood glucose‐lowering effect (Figure 4B) and a correspondingly high area under the curve (AUC) (Figure 4C) after subcutaneous (s.c.) administration of PBS or FA1 alone, with BGL remaining >300 mg/dL. Administration of insulin alone produced a rapid (hypoglycemic) but short‐lived drop in BGL, which began to rise 6 h later (Figure 4B). However, the BGL of diabetic rats in the FA1+insulin (5 mg/mL) group decreased continuously, reaching a low point at 60 h, then rebounded to a high BGL (>200 mg/dL) at 66 h post‐injection. This group exhibited a marked, sustained reduction in BGL and a high AUC, maintaining normoglycemia for a longer period than the controls. Interestingly, Lantus maintained normoglycemia for up to ∼18 h, with a low AUC (Figure 4B,C), whereas the hydrogel formulation extended normoglycemic control to nearly 60 h. The sustained release and glucose‐level lowering were further supported by the sustained maintenance of serum insulin levels, as measured with an insulin ELISA kit. The data showed insulin levels remained elevated for 60 h before declining (Figure 4D) for the insulin‐encapsulated hydrogel group. On the contrary, it was very high at 0.5 h, leading to hypoglycemia in the only insulin‐treated group of rats. In the Lantus‐administered group, serum insulin levels were sustained until 18 h, after which they were minimal (Figure 4D). Thus, consistent with the observed glycemic profile, serum insulin measurements revealed a prolonged systemic presence of insulin, indicating controlled and sustained release from the hydrogel matrix. The temporal profiles of insulin and glucose levels showed an inverse relationship, with increased circulating insulin associated with a progressive decline in BGL (Figure S8). Despite the long‐term glycemic control, the present formulation should be recognized as a finite insulin reservoir. The decline in serum insulin levels after ∼60 h, along with a rebound in BGL, indicates depletion of the available insulin reservoir and suggests that the formulation may not provide constant steady‐state insulin exposure indefinitely.

**FIGURE 4:**
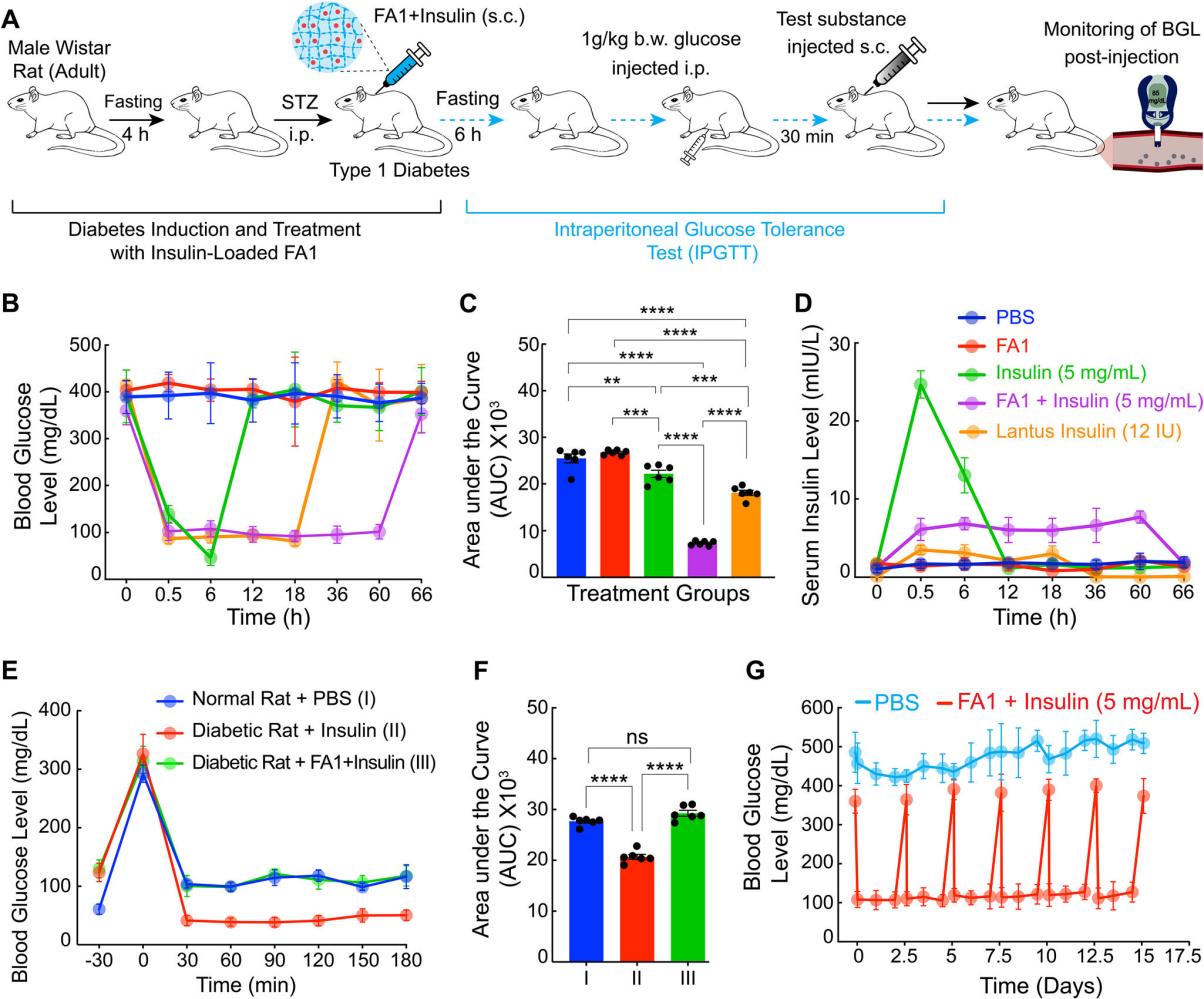
In vivo therapeutic efficacy of the insulin‐loaded FA1 amyloid hydrogel in STZ‐induced Type 1 diabetic rats. (A) Schematic representation of the experimental design. Type 1 diabetes was induced in male Wistar rats by i.p. administration of STZ, followed by s.c. administration of the insulin (denoted by red circles)‐loaded FA1 hydrogel to evaluate its long‐acting hypoglycemic effect. For the IPGTT, fasted rats received an intraperitoneal glucose challenge (1 g/kg b.w.), followed 30 min later by subcutaneous administration of the test formulation, and BGLs were monitored at indicated time points. One of the icons has been generated using BioRender.com. (B) Temporal changes in BGLs following a single subcutaneous administration of the different formulations, demonstrating prolonged glycemic regulation by the FA1+insulin formulation. (C) AUC analysis of the blood glucose profiles shown in B, comparing the overall glycemic response among the treatment groups. (D) Serum insulin concentrations measured at the indicated time points following treatment, illustrating sustained systemic insulin availability from the hydrogel formulation. (E) Blood glucose profiles obtained during the IPGTT, demonstrating improved glucose clearance and prolonged glycemic control in rats treated with the FA1+insulin formulation compared with the control groups. (F) Corresponding AUC analysis of the IPGTT blood glucose curves shown in E, indicating the significance of our formulation. (G) Long‐term blood glucose profiles of diabetic rats following repeated subcutaneous administration of the FA1+insulin formulation every 2.5 days for 15 days, demonstrating sustained therapeutic efficacy throughout the treatment period. For C and F, the data represent the mean ± S.E.M. (*n* = 6), and the statistical significance was determined using a one‐way ANOVA, followed by a Tukey's multiple‐comparison test [^****^ *p* < 0.0001, ^***^ *p* < 0.0002, ^**^ *p* < 0.0021 and ^ns^ *p* > 0.1234 with a 95% confidence interval]. Data were plotted and analyzed using GraphPad Prism v9.5.1, and figures were prepared and assembled using Adobe Illustrator 2023 v27.0.

Owing to the impressive therapeutic effect of FA1+Insulin, an intraperitoneal glucose tolerance test (IPGTT) was subsequently performed in glucose‐treated diabetic rats (*n* = 6) to assess the dynamic changes in BGL due to our formulation (Figure 4A). Upon glucose injection, the BGLs of rats treated with FA1+insulin rose rapidly within the first 30 min but returned to the initial normal state within the next 30 min, matching those of healthy rats, and simultaneously demonstrating a comparable AUC (Figure 4E,F). In contrast, only insulin‐treated diabetic rats were unable to maintain their BGLs within the normal range and showed hypoglycemia, with a corresponding low AUC (Figure 4E,F). These results demonstrate that the FA1+insulin formulation responds well to glucose in vivo, owing to its sustained insulin release. Therefore, the improved glucose tolerance observed during the IPGTT, along with reduced AUC values, further supports the hydrogel system's ability to maintain physiologically relevant insulin levels and facilitate efficient glucose clearance.

To verify the consistent and safe euglycemic effect of our formulation, we repeatedly administered it for 15 days (one injection every 2.5 days). As shown in Figure 4G, a single subcutaneous injection was sufficient to maintain a normal BGL for 2.5 days, and the injection was repeated for 15 days without risk of hypoglycemia, demonstrating sustained glycemic control over an extended period with no evidence of diminished efficacy and highlighting the robustness of the depot system. However, the BGL fluctuations observed during repeated administration highlight the glycemic variability associated with the current release and dosing profile, underscoring the need to further optimize insulin loading, release kinetics, and dosing intervals to achieve more uniform and predictable glycemic control. Collectively, these findings establish that peptide‐based functional amyloid hydrogels may serve as an effective subcutaneous long‐term insulin depot, enabling sustained glycemic control for several days, reducing injection frequency, minimizing injection‐site complications, and improving patient compliance.

### 2.5 Cross‐Seeding Assessment and Long‐Term Biosafety Evaluation of the Amyloid Hydrogel Formulation

As functional amyloid‐based biomaterials continue to emerge as promising platforms for therapeutic delivery, establishing that they do not nucleate the aggregation of disease‐associated amyloidogenic proteins has become a critical prerequisite for their therapeutic application [29, 64, 65]. Because heterologous amyloid cross‐seeding has been implicated in the propagation of protein misfolding [32, 65], evaluation of our peptide‐based functional amyloid hydrogel's ability to promote amylin/IAPP (associated with Type 2 diabetes) [66], and α‐Syn (associated with Parkinson's disease) aggregation is essential to demonstrate its biosafety [61]. Thus, given the relevance of IAPP as a diabetes‐associated amyloid protein [66], we evaluated the heterologous cross‐seeding potential of FA1 amyloid fibrils toward IAPP. The ThT kinetics (Figure 5A), corresponding lag times (Figure S9A), and far‐UV CD spectroscopy (Figure S9B) analyses showed no detectable acceleration or alteration in IAPP aggregation in the presence of FA1 amyloid fibrils, indicating the absence of heterologous cross‐seeding under the tested conditions.

**FIGURE 5:**
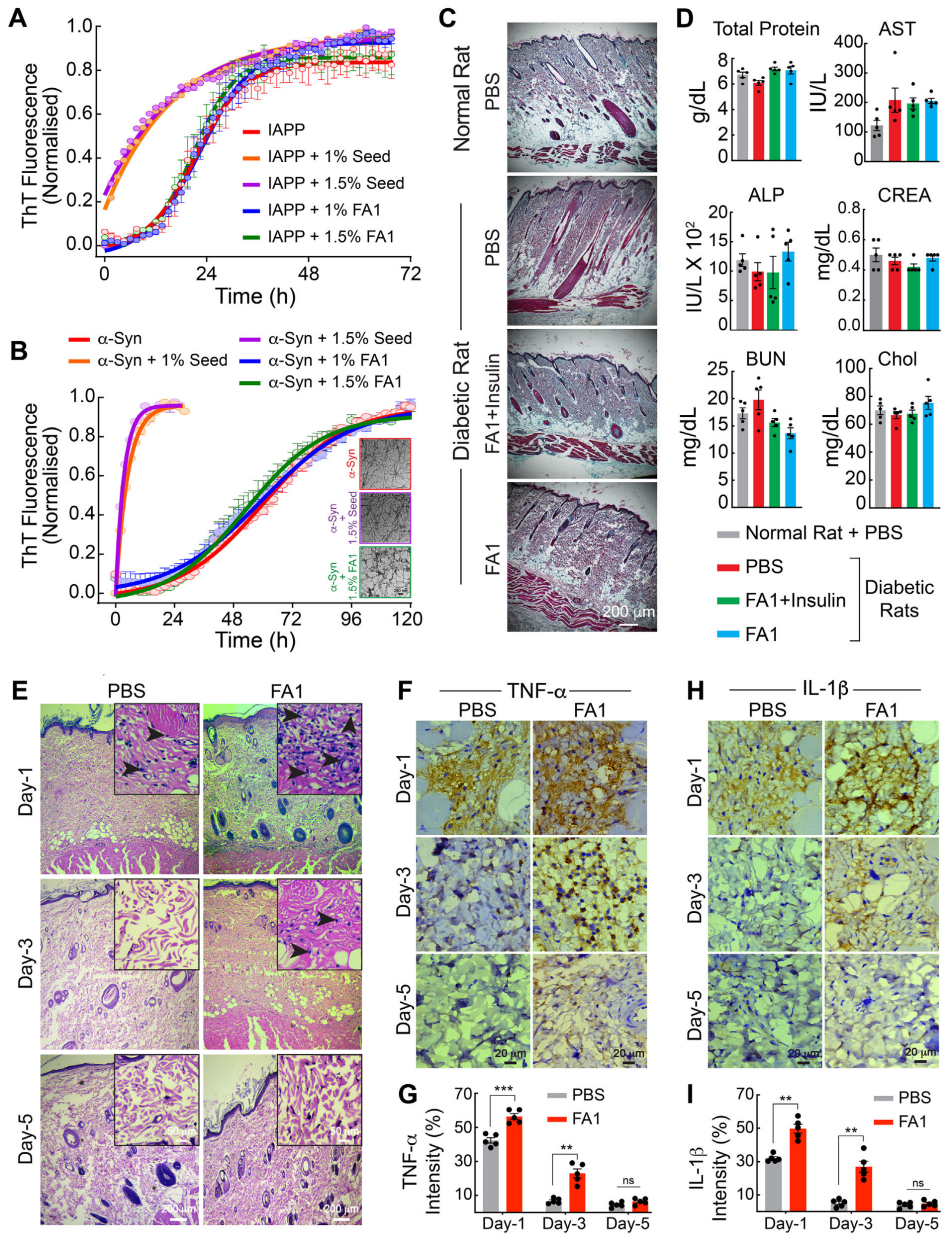
Amyloid cross‐seeding and long‐term biosafety evaluation of the FA1 amyloid hydrogel following repeated administration for 15 days. (A and B) Assessment of the heterologous cross‐seeding propensity of the FA1 amyloid hydrogel towards WT IAPP (A) and α‐Syn (B), compared with preformed IAPP and α‐Syn fibril seeds (positive control for homologous seeding), by monitoring ThT fluorescence at 37°C, indicating that FA1 hydrogel seeds did not accelerate IAPP or α‐Syn amyloid formation under identical experimental conditions. ThT fluorescence (480 nm) was monitored at regular intervals, and the normalized fluorescence intensities were plotted as a function of incubation time for each experimental group. The inset in (B) shows representative TEM images of endpoint samples comparing WT α‐Syn following heterologous incubation with 1.5% FA1 hydrogel fibrils and homologous seeding with 1.5% α‐Syn preformed fibril seeds. (C) Representative MT staining images of the skin injection sites from the four treatment groups, showing collagen deposition following repeated subcutaneous administration. (D) Serum biochemical analyses of liver and kidney function parameters following repeated administration every 2.5 days for 15 days. The treatment groups comprised healthy rats receiving subcutaneous PBS (Group 1), STZ‐induced diabetic rats receiving PBS (Group 2), insulin‐loaded FA1 hydrogel (FA1+insulin; Group 3), and FA1 hydrogel alone (Group 4), demonstrating no evidence of systemic toxicity. (E) Representative H/E staining of the injection sites showing the local tissue response following repeated hydrogel administration. (F, H) Representative immunohistochemical staining images of the inflammatory cytokines TNF‐α (F) and IL‐1β (H) at the indicated time points following administration. (G, I) Quantitative analysis of TNF‐α (G) and IL‐1β (I) immunostaining, demonstrating a transient local inflammatory response that progressively resolved over time. For D, G, and I, the data represent the mean ± S.E.M. (*n* = 5). Statistical significance was determined using a one‐way ANOVA followed by Dunnett's multiple‐comparisons test (D), or a two‐tailed unpaired t‐test with Welch's correction (G, I) [^****^ *p* < 0.0001, ^***^ *p* < 0.0002, ^**^ *p* < 0.0021, and ^ns^ *p* > 0.1234 with a 95% confidence level]. Data were plotted and analyzed using GraphPad Prism v9.5.1 and ImageJ v1.54 (NIH, Bethesda, USA), as applicable, and figures were prepared and assembled using Adobe Illustrator 2023 v27.0.

Further, to investigate whether our amyloid hydrogel can template the aggregation of one of the brain‐related pathological proteins, α‐Syn, we monitored fibrillation kinetics using the ThT fluorescence assay, which enabled real‐time detection of amyloid β‐sheet formation. Wild‐type (WT) α‐Syn was chosen as a stringent model because of its intrinsic propensity to undergo amyloid fibrillation and its reported susceptibility to heterologous amyloid‐mediated nucleation [65, 67], making it a sensitive probe for assessing this phenomenon. As expected, the addition of 1% and 1.5% preformed α‐Syn fibril seeds (Figure S9C) markedly shortened the lag phase and accelerated aggregation kinetics, confirming efficient homologous seeding (Figure 5B). In contrast, the presence of 1% and 1.5% FA1 fibril seeds did not alter the aggregation profile of WT α‐Syn, with lag times that were approximately the same (Figure S9D), indicating that the hydrogel does not promote cross‐seeding under the tested conditions (Figure 5B). As per its intrinsically disordered nature, far‐UV CD analysis of WT α‐Syn exhibited random coil conformation at day 0, whereas results after incubation revealed a seeding‐mediated transition to a β‐sheet‐rich structure only in the presence of homologous α‐Syn seeds, as evidenced by increased ellipticity at 218 nm; in contrast, FA1 hydrogel fibrils failed to induce this conformational transition, indicating the absence of heterologous seeding (Figure S9E). This was further confirmed by TEM imaging, and the results were consistent with the CD analysis (Inset, Figure 5B; Figure S9F). Collectively, the ThT, CD, and TEM analyses demonstrate that although preformed α‐Syn seeds efficiently catalyze α‐Syn aggregation, our amyloid hydrogel, FA1, was unable to seed/initiate pathogenic α‐Syn fibrillation.

To evaluate the therapeutic safety of the formulation, we conducted a long‐term in vivo biosafety study involving six repeated injections (Figure S10A). Insulin‐loaded FA1 amyloid hydrogel promoted progressive body weight gain (Figure S10B) and reduced water intake in diabetic rats (Figure S10C), patterns consistent with those of normal healthy rats. Histological (Hematoxylin and Eosin (H/E) and Masson's Trichrome (MT)) staining analyses revealed no tissue damage (in six major body organs) or abnormal collagen deposition at the injection site (Figure 5C; Figures S10D and S11). Furthermore, hematological parameters and serum biomarkers of hepatic [aspartate aminotransferase (AST)] and renal [blood urea nitrogen (BUN)] function remained comparable to those in controls, indicating that repeated administration did not induce systemic toxicity or organ dysfunction (Figure 5D; Figure S12). Next, we assessed local tissue responses following subcutaneous administration of the FA1 hydrogel using H/E staining and further confirmed them by immunohistochemistry (IHC) for TNF‐α and IL‐1β. Both PBS and FA1 hydrogel groups exhibited transient acute inflammation characterized by leucocyte infiltration and elevated cytokine expression on Day 1. While the inflammatory response resolved more rapidly in the PBS group, the FA1 hydrogel group showed a modestly prolonged yet well‐controlled response that subsided by Day 5, with no evidence of tissue damage or necrosis (Figure 5E–I). This suggests that the amyloid hydrogel elicits a transient, self‐resolving inflammatory response, underscoring its favorable biocompatibility for subcutaneous applications. Collectively, these findings demonstrate that the insulin‐encapsulated amyloid hydrogel is a biocompatible, well‐tolerated, long‐acting, biosafe formulation that causes no overt tissue or organ toxicity and holds strong therapeutic potential for sustained diabetes therapy.

### 2.6 Modulation of Functional Insulin Release by the Amyloid Hydrogels Incorporating Bioadditives

We hypothesized that the release properties of amyloid hydrogels could be rationally tuned by incorporating biocompatible additives without disrupting the structural integrity of the underlying amyloid fibrillar network, thereby extending insulin release and further reducing injection frequency. As a proof of concept, we selected S.A. as a representative bio‐additive to demonstrate this strategy [54, 68], which has been reported to modulate the biophysical characteristics of the drug‐delivery depot [68, 69, 70, 71, 72], although the approach is expected to be readily adaptable to other biocompatible additives to tailor release kinetics.

Thus, along these lines, we fabricated composite hydrogels by incorporating three concentrations of S.A. (FA1+S.A.; Figure 6A). A traditional gel inversion test was performed to confirm their formation (Figure 6B). Their TEM, AFM, and Cryo‐SEM imaging revealed the retention of the characteristic dense fibrillar morphology and porous architecture (Figure 6C–E; Figure S13A). Their FTIR spectroscopy exhibited the retention of the characteristic cross‐β‐sheet signatures (Figure S13B), demonstrating that the incorporation of S.A. preserves the molecular architecture and intermolecular hydrogen‐bonding network of the amyloid fibrils without disrupting their amyloidogenic structure. ThT and CR‐binding assays confirmed that all composite hydrogels retained the canonical amyloid signatures, evidenced by strong ThT fluorescence (Figure S13C) and the typical CR red shift (Figure 6F). Although CR absorbance decreased with increased alginate concentration, indicating a lower relative fibrillar content, the preserved amyloid signatures indicate that alginate incorporation does not disrupt the amyloidogenic nature of the peptide network. Rheological analyses further demonstrated that all composite hydrogels remained viscoelastic (G′ > G′′) and exhibited shear‐thinning (Figure 6G; Figure S13D). While the 0.3% and 0.6% S.A. formulations exhibited rapid and reproducible thixotropic recovery, the 0.9% S.A. hydrogel showed impaired network reconstruction after shear, thereby identifying the 0.6% S.A. composite hydrogel as the optimal composition for sustained drug‐delivery applications.

**FIGURE 6:**
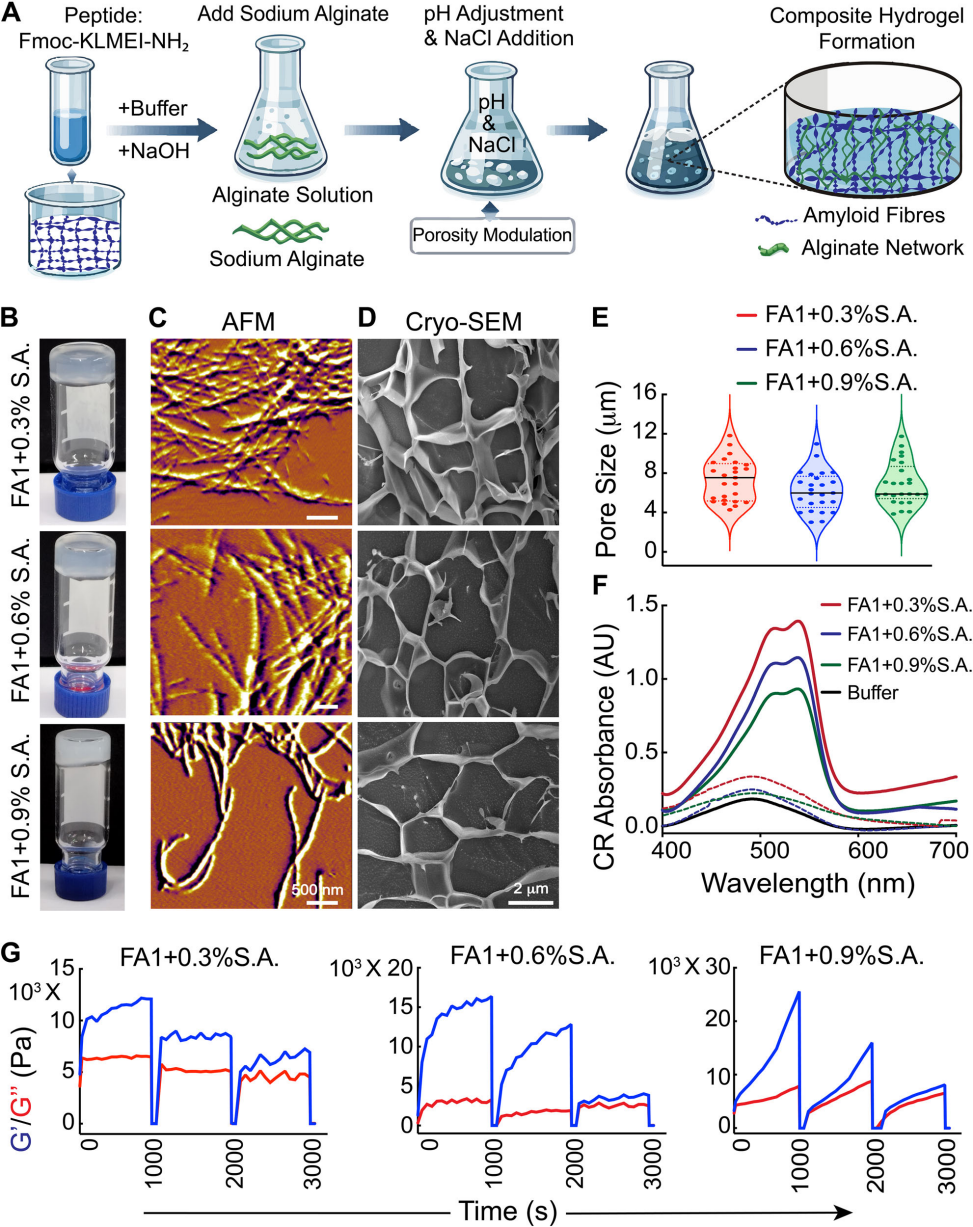
Preparation and biophysical characterization of the peptide‐inspired functional amyloid‐sodium alginate composite hydrogels. (A) Schematic illustration of the fabrication of peptide‐based functional amyloid‐S.A. composite hydrogels containing different concentrations of sodium alginate. (B) Representative gel inversion test confirming successful formation of the composite hydrogels. (C) Atomic force micrographs of the composite hydrogels, depicting their characteristic nanofibrillar architecture, forming an interconnected mesh‐like framework. The scale bar is 500 nm. (D) Cryo‐scanning electron micrographs of the composite hydrogels, showing their porous three‐dimensional microstructure. The scale bar is 2 µm. (E) Violin plots showing the pore size distribution of the composite hydrogels quantified from Cryo‐SEM images. (F) CR binding assay demonstrating the characteristic increase in absorbance accompanied by a bathochromic shift in the absorption maximum, confirming preservation of the amyloid architecture following S.A. incorporation. The corresponding peptide monomers are shown as controls (—). (G) Step‐strain rheological analysis at 37°C demonstrating reversible gel‐sol transitions and rapid recovery of the storage modulus following repeated high‐ and low‐strain cycles, indicative of the thixotropic self‐healing behavior of the composite hydrogels. Data were plotted and analyzed using GraphPad Prism v9.5.1 and ImageJ v1.54 (NIH, Bethesda, USA), as applicable, and figures were prepared and assembled using Adobe Illustrator 2023 v27.0.

To evaluate their suitability as injectable sustained‐release depots, the composite hydrogels were systematically characterized for swelling, biocompatibility, and insulin delivery. All formulations exhibited minimal swelling (∼10% after 24 h), high cytocompatibility (>90% NIH 3T3 viability), and excellent hemocompatibility, demonstrating their potential usage for biomedical applications (Figure 7A,B; Figure S14A). FITC‐insulin was efficiently encapsulated, as evidenced by >95% EE (Figure S14B) and the presence of α‐helical secondary conformation, as evidenced in FTIR spectroscopy (Figure S14C). Moreover, all formulations retained gel integrity at insulin concentrations up to 7.5 mg/mL (Figure 7C).

**FIGURE 7:**
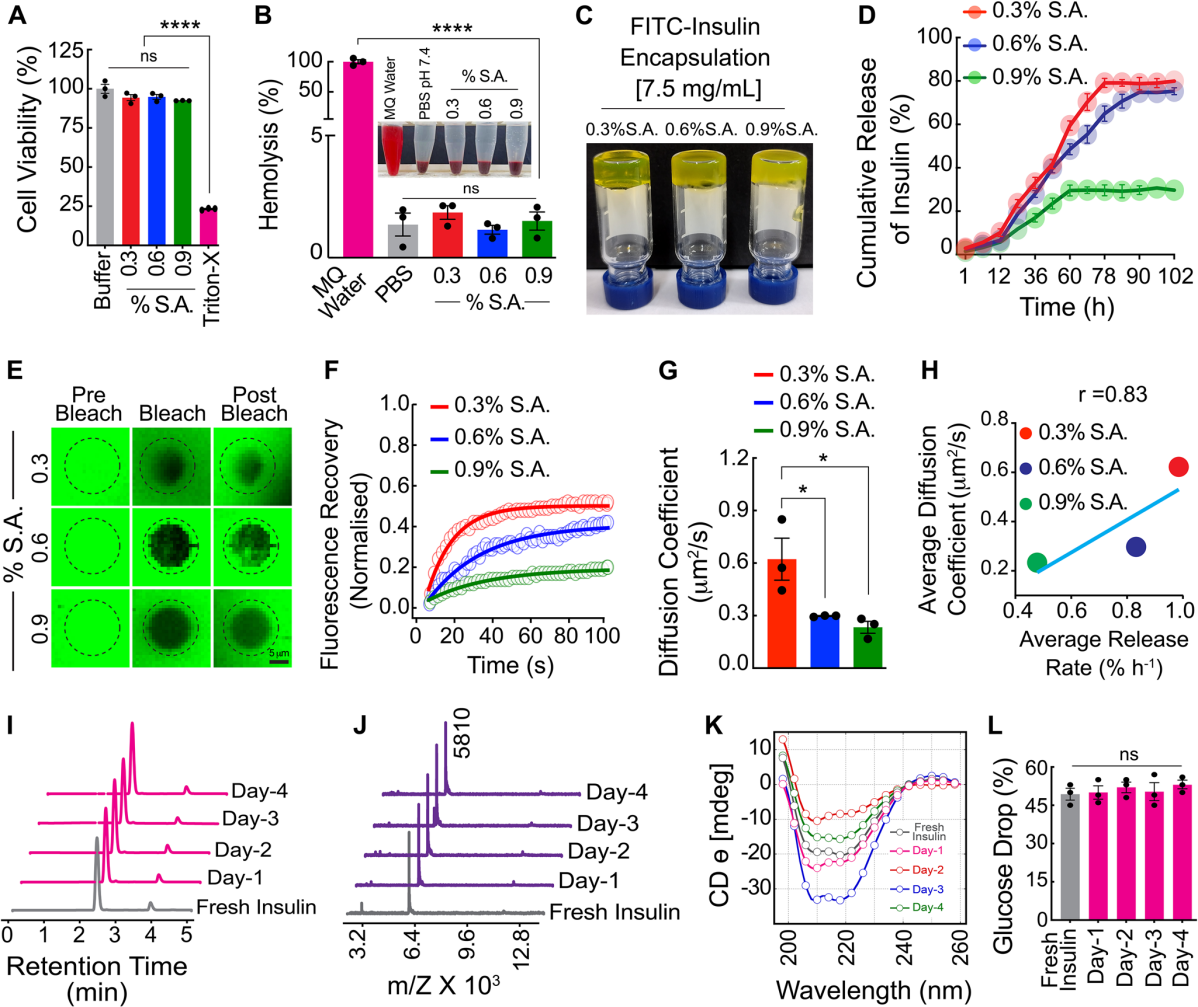
Functional evaluation of peptide‐based functional amyloid‐sodium alginate composite hydrogels for sustained insulin delivery. (A) Cytocompatibility of composite hydrogels containing different concentrations of S.A. (0.3%, 0.6%, and 0.9%) for 24 h of incubation, evaluated by MTT assay. (B) Hemocompatibility of these composite hydrogels was assessed by hemolysis assay. Representative photographs of the assay solutions (inset) and the corresponding % hemolysis quantification demonstrate minimal erythrocyte lysis after incubation with the composite hydrogels. (C) Representative inverted‐tube images of insulin‐loaded composite hydrogels containing 7.5 mg/mL FITC‐insulin, confirming successful gel formation following insulin encapsulation. (D) Cumulative in vitro insulin release profiles from the composite hydrogels over 102 h, demonstrating sustained and tunable release kinetics. (E) Representative images of the FRAP study using FITC‐insulin‐encapsulated composite hydrogel, depicting pre‐bleach, bleach (0 s), and post‐bleach phases. (F) Normalized fluorescence recovery curves, showing moderate to low recovery. It shows insulin diffusion within the composite hydrogel matrices. (G) Quantitative analysis of the FRAP experiments showing the calculated diffusion coefficients of FITC‐insulin. (H) Scatter plot showing a direct relationship between the FRAP‐derived insulin diffusion coefficient and average release rate for 0.3%, 0.6%, and 0.9% S.A. composite hydrogels, with a correlation coefficient of 0.83. (I‐L) Structural and functional stability of insulin released from the lead FA1+0.6% S.A. composite hydrogel following sustained release at 37°C for up to 4 days. (I) IP‐HPLC chromatograms comparing freshly prepared and composite hydrogel‐released insulin. (J) MALDI‐TOF mass spectra confirming preservation of the molecular mass of the composite hydrogel‐released insulin. (K) Far‐UV CD spectra showing retention of the native α‐helical secondary structure following sustained release. (L) In vivo functionality assay in STZ‐induced Type 1 diabetic rats, confirming that the released insulin retains its glucose‐lowering activity. For A, B, G, and L, the data represent the mean S.E.M. (*n* = 3), and the statistical significance was determined using a one‐way ANOVA, followed by Dunnett's (A and L) or Tukey's multiple‐comparisons test (B, and G) [^****^ *p* < 0.0001, ^***^ *p* < 0.0002, ^**^ *p* < 0.0021, ^*^ *p* < 0.0332 and ^ns^ *p* > 0.1234 with a 95% confidence interval]. For simplicity, the composite hydrogel formulations FA1 + 0.3% S.A., FA1 + 0.6% S.A., and FA1 + 0.9% S.A. are denoted as 0.3% S.A., 0.6% S.A., and 0.9% S.A., respectively, throughout the figure. Data were plotted and analyzed using GraphPad Prism v9.5.1, OriginPro 2021 v9.8.0, KaleidaGraph v4.03, and ImageJ v1.54 (NIH, Bethesda, USA), as applicable, and figures were prepared and assembled using Adobe Illustrator 2023 v27.0.

In vitro release studies showed that S.A. incorporation modulated insulin release, with the FA1+0.3% and FA1+0.6% S.A. composite hydrogels providing sustained release for ∼78 h and ∼90 h, respectively, whereas the FA1+0.9% S.A. formulation exhibited markedly reduced cumulative release (∼30%) (Figure 7D). Consistently, the FA1+0.3% and FA1+0.6% S.A. hydrogels followed near‐zero‐order release kinetics, while the FA1+0.9% S.A. formulation transitioned to diffusion‐dominated Higuchi kinetics [62] (Table S2). We further performed FRAP analysis, which demonstrated progressively restricted insulin mobility with increasing alginate content, reflected by reduced fluorescence recovery, longer half‐times, and lower diffusion coefficients, with the FA1+0.6% S.A. formulation providing the optimal balance between molecular mobility and sustained release (Figure 7E–G; Figure S14D,E). Although the Cryo‐SEM analysis (Figure 6E) revealed comparable pore‐size distributions across the composite hydrogels, correlation analysis between the FRAP‐derived insulin diffusion coefficient and average release rate for FA1+0.3%, FA1+0.6%, and FA1+0.9% S.A. composite hydrogels demonstrated a strong positive relationship (*r* = 0.83) (Figure 7H). Also, the FA1+0.9% S.A. formulation exhibited highly restricted release, potentially due to altered insulin‐matrix interactions and/or increased insulin binding, both of which require further investigation. Collectively, these results indicate that S.A. incorporation enables tunable modulation of insulin release through changes in insulin mobility and the physicochemical properties of the composite network rather than pore‐size variation alone.

Because sustained protein delivery requires preservation of therapeutic activity throughout the release period, the stability of insulin released from the lead FA1+0.6% S.A. composite hydrogel was evaluated at 37°C for 4 days. IP‐HPLC, MALDI‐TOF MS, and far‐UV CD analyses confirmed that the released insulin retained its chemical integrity, primary structure, and native secondary structure without detectable degradation, while in vivo glucose‐lowering studies demonstrated retention of chemical/structural activity comparable to fresh insulin (Figure 7I–L). Collectively, these findings identify the FA1+0.6% S.A. composite hydrogel as the optimal and promising protein delivery platform and support its further in vivo evaluation for prolonged glycemic control.

## 3 Discussion

Amyloids are historically associated with protein misfolding and aggregation disorders such as Alzheimer's and Parkinson's diseases [32, 36, 61, 73]. However, the discovery of functional amyloids over the last two decades of research has fundamentally challenged the view that amyloids can perform normal biological functions in the host organism [29, 31, 34, 41, 74, 75]. Furthermore, the designed amyloids have been shown to be broadly applicable in diverse biotechnological applications, encompassing regenerative medicine, tissue engineering, and drug delivery [34, 43, 44, 47, 48, 49, 50, 51, 55, 76]. Recent demonstrations that dairy protein‐derived amyloid fibrils are safe following oral administration further support the concept that amyloid toxicity is not universal but depends on the molecular sequence, structure, and surface properties of these fibrils [37, 39]. Their exceptional structural stability against harsh physicochemical conditions, together with growing evidence demonstrating the safety of naturally occurring amyloids, has inspired the development of engineered amyloid‐based biomaterials [16, 29]. In particular, the protective role of the growing embryo against harsh environmental conditions, mediated by functional chorion amyloids in the silkworm [35], motivated us to hypothesize that an amyloid fibrillar network could simultaneously stabilize therapeutic proteins and serve as a sustained‐release depot (Figure S15). In this context, the widespread clinical use of therapeutic proteins is limited by rapid diffusion, enzymatic degradation, and structural instability following administration, necessitating repeated dosing to maintain therapeutic efficacy [1]. To address this, we demonstrate that peptide‐based functional amyloid hydrogels can serve a dual purpose, not only as sustained‐release depots but also as protein‐stabilizing biomaterials. Consistent with this hypothesis, we demonstrated that insulin encapsulated within the amyloid hydrogel retained its primary structure, native secondary conformation, and biological activity during long‐term storage (6 months of our evaluation) at 4°C and during prolonged release at 37°C (physiological temperature). These findings indicate that the amyloid fibrillar network functions as more than a passive reservoir; instead, it stabilizes the therapeutic cargo while permitting diffusion‐governed release (Figure 3). Such dual functionality distinguishes functional amyloid hydrogels from many conventional depot systems, which primarily regulate release kinetics without directly contributing to protein stability [77, 78].

The data demonstrated sustained release of structurally intact and biologically active insulin for up to 72 h at 37°C and prolonged therapeutic efficacy in vivo, maintaining normoglycemia for ∼2.5 days following a single administration. This is also consistent with previous reports demonstrating sustained delivery of bioactive proteins (growth factors) from functional amyloid hydrogels [47, 48]. The prolonged in vivo efficacy observed here compares favorably with many reported injectable insulin depots, which often suffer from burst release, limited protein stability, complex fabrication, or insufficient control over long‐term release kinetics [1, 10, 77, 79]. Unlike insulin pumps [80] or repeated injections, which rely on frequent dosing and patient adherence, our platform provides a single injectable depot that delivers sustained protein stabilization and release. Beyond improving patient compliance, reducing injection frequency may also decrease repeated local tissue trauma and potentially lower the risk of injection‐site complications, including the rare occurrence of insulin‐derived amyloidosis associated with repeated injections at the same site [12, 81]. More broadly, these findings establish diabetes as a representative disease model and insulin as a representative protein cargo, demonstrating that functional amyloid hydrogels can serve as a versatile injectable depot for sustained delivery of therapeutic proteins. Given that many biologics require repeated administration due to rapid clearance and limited stability, this platform may be readily extended to other protein‐ and peptide‐based therapeutics.

A major consideration for the therapeutic application of functional amyloid biomaterials is their biosafety, particularly their potential to cross the blood‐brain barrier (BBB) and, in turn, to induce heterologous amyloid cross‐seeding in the brain and other parts of the body, which has been implicated in the initiation and/or aggravation of protein misfolding diseases [64, 65]. For example, both Tau and Aβ fibrils have been reported to accelerate α‐Syn aggregation [82, 83]. Further, since peptide‐based functional amyloid fibrils or their degradation products may reach and interact with the CNS if they cross the BBB, their permeability is a critical biosafety consideration. In our study, following subcutaneous administration, the intact hydrogel forms a relatively large, localized supramolecular fibrillar network at the injection site rather than a freely circulating amyloid seed. Our previous studies have demonstrated that this short‐peptide‐based amyloid hydrogel can degrade over time [45]. Importantly, amyloid fibrils may not be considered a uniform class with respect to BBB transport. Although certain pathological amyloid seeds or protein aggregates have been reported to disseminate to the CNS under specific pathological conditions [84], such behavior is highly dependent on the physicochemical properties and biological nature of the particular amyloid species and may not be generalized to all amyloid‐based materials [30, 85]. Furthermore, the BBB is highly selective, and the entry of circulating macromolecules or protein aggregates generally requires specific transport mechanisms, such as receptor‐mediated transcytosis, and is strongly dependent on the material's physicochemical properties and surface characteristics [86].

Furthermore, amyloid formation is directly relevant to diabetes, as the aggregation and deposition of IAPP are major pathological features associated with pancreatic islet dysfunction, particularly in Type 2 diabetes [66]. In addition, several other amyloidogenic proteins have been implicated in diabetes and its secondary complications, including Amyloid‐β in diabetes‐associated neurodegenerative pathology [87] and α‐Syn in the context of metabolic dysfunction and Parkinsonian pathology [88, 89], although the mechanistic relationship remains incompletely understood. Also, diabetes and impaired insulin signaling have been associated with altered brain insulin function, neuroinflammation, oxidative stress, and dysregulated amyloid‐β metabolism, providing a mechanistic link between metabolic dysfunction and neurodegenerative amyloid pathology [87, 90]. Given the broader association between diabetes and multiple amyloidogenic proteins, we further assessed the heterologous seeding potential of our functional amyloid fibrils against both IAPP, the diabetes‐associated amyloid protein, and α‐Syn, a pathological amyloidogenic protein implicated in neurodegeneration, and observed no detectable heterologous cross‐seeding activity under the experimental conditions tested. Importantly, amyloid seeding is not a generic property of all amyloid assemblies but depends on molecular recognition and structural compatibility between the seed and substrate, including sequence and conformational homology [37, 39]. Therefore, the absence of detectable heterologous cross‐seeding with IAPP and α‐Syn indicates that our functional amyloid fibrils do not exhibit generic seeding activity toward this disease‐associated amyloid protein under the tested conditions. Thus, the amyloid nature of the present fibrils does not establish their ability to cross the BBB. However, because the biodistribution and BBB permeability of the intact hydrogel, its fibrillar components, and their degradation products were not directly evaluated in the present study, the ability of these components to reach or cross the BBB requires further investigation to establish the CNS safety profile of this amyloid hydrogel platform. Therefore, together with the absence of systemic toxicity and only a transient, self‐resolving local inflammatory response to the hydrogel's administration, these findings demonstrate the favorable safety profile of the peptide‐based functional amyloid hydrogels.

Our previous studies have shown that the release kinetics of amyloid hydrogels can be tuned either through rational peptide design or by incorporating external modulators that alter the network architecture and pore size [46]. For example, the addition of NaCl has been reported to regulate growth factor release from peptide‐based amyloid hydrogels, thereby influencing neural stem cell differentiation [47]. We hypothesized that incorporating biocompatible bioadditives may further tune the amyloid hydrogel network and may act as a higher sustained‐release system. Using sodium alginate as a representative bioadditive [54], we demonstrated that the release profile of structurally intact and biologically active insulin can be extended while preserving the hydrogel's injectability and biocompatibility, supporting the future development of very long‐acting insulin formation. Such a long‐acting insulin formulation may be advantageous for patients in the early stage of diabetes, where sustained low‐dose insulin supplementation may help glycemic control. Taken together, these findings establish peptide‐based functional amyloid hydrogels as a tunable, scalable, and versatile biomaterial platform that integrates protein stabilization with tunable sustained release.

While our drug depot has numerous advantages, it also presents a few disadvantages. For example, these peptide‐based functional amyloid hydrogels are not glucose‐responsive, thus cannot form a closed‐loop insulin delivery system, and lack physiological mimicry. Moreover, while FA1 amyloid hydrogel provides glucose control for up to 2.5 days, other injectable delivery platforms for insulin and other biologics demonstrated longer therapeutic release windows. However, the sustainable release can be further tuned using bioadditives, as evidenced by our data with sodium alginate. Also, detailed, longer‐term pharmacokinetics and pharmacodynamics studies are required in the future to further establish the therapeutic potential of this platform. However, their animal‐free composition, cross‐linker‐free fabrication, biodegradability, and straightforward, cost‐effective synthesis from short peptides further support their potential for next‐generation depot formulation for therapeutic proteins/polypeptides.

## 4 Experimental Section

We used all the chemicals as supplied; no further purification was performed. 1‐Hydroxybenzotriazole hydrate (HOBt) (Cat No. 157260), Triisopropylsilane (TIPS) (Cat No. 233781), Trifluoroacetic acid (TFA) (Cat No. T6508), and N, N’‐Diisopropylcarbodiimide (DIC) (Cat No. D4781) were purchased from Sigma (USA). N, N‐Dimethylformamide (DMF) (Cat No. 8.22275.2521), Dichloromethane (DCM) (Cat No. 1.94508.2521), Acetonitrile (ACN) (Cat No. 60003025001730), and Diethyl ether (Cat No. 1.07026.0521) were purchased from Merck Millipore. Wang resin (100‐200 mesh, 0.7 mmol/ g) (Cat No. 8.55002), and 4‐Dimethylamino pyridine (DMAP) (Cat No. 8.51055) were purchased from Novabiochem (Germany). Human recombinant insulin and insulin glargine (Lantus) were procured from Sigma‐Aldrich (St. Louis, MO, USA) and Sanofi India Limited, respectively. Color‐coded Pre‐stained Protein Marker, Low Range (1.7‐42 kDa) was procured from Cell Signaling Technology.

### 4.1 Synthesis and Purification of Fmoc‐Peptides

Peptide synthesis was performed using a manual solid‐phase peptide synthesis method with 9‐fluorenylmethyloxycarbonyl (Fmoc) chemistry [91]. The synthesis was executed on a 0.25 mmol scale on the basis of the resin's loading capability (∼0.25 mmol). The conjugation reactions were achieved by attachment of the first amino acid to the resin in the presence of DMAP, Fmoc‐protected amino acid (1 eq.), and DIC and HOBt in DMF (1 eq.). Next, the mixture was left to incubate at RT for 4–5 h, after which the Fmoc protection group was eliminated using piperidine (25%) in DMF. In the end, a cleavage mixture of 0.88:0.05:0.05:0.02 of TFA: water: phenol: TIPS was utilized to cut the carboxyl‐terminal group off the wang resin. Finally, the peptide was made to precipitate in chilled diethyl ether, and the crude peptide pellet was isolated using centrifugation at 8500 r.p.m. at 4°C for 15 min.

Further, the crude peptide was resuspended in 50% (v/v) ACN for purification by the RP‐HPLC method. Preparative RP‐HPLC was executed on a Shimadzu LC20‐AR instrument equipped with a Shimadzu C18 column (250 mm long × 10 mm diameter, 5 µm particle size) with a UV/vis detector at a specific wavelength (254 nm). The eluents were 0.1% TFA in water and 0.1% TFA in ACN, which was used as a gradient mobile phase. Initially, the ACN content was increased from 0% to 90% over 20 min, then decreased linearly to 0% over 10 min at a constant flow rate (4 mL/min). The peptide fractions were subsequently collected and lyophilized to produce a white solid powder. The sequences of the synthesized peptides were verified by analytical HPLC and ESI‐MS analyses.

### 4.2 Preparation of Amyloid Hydrogels

Amyloid hydrogels [15 mg/mL] [Fmoc‐KLMEI (Fmoc‐Lys‐Leu‐Met‐Glu‐Ile) and Fmoc‐KLLDI (Fmoc‐Lys‐ Leu‐Leu‐Asp‐Ile), designated later as FA1 and FA2, respectively] were formed by dissolving the synthesized peptides in 20 mm Phosphate buffer (PB), pH 7.4, following the previously reported protocols [45, 46, 47, 48]. Briefly, 7.5 mg of each peptide was first resuspended in 500 µL of PB (20 mm), pH 7.4, then dissolved by raising the pH to 10 with 6N NaOH. 300 mm NaCl was then added, and 6N HCl was used to adjust the pH to 7.4 to induce gelation. Henceforth, 15 mg/mL of both hydrogels was used in the studies, unless otherwise mentioned.

### 4.3 Preparation of Composite Hydrogels with S.A

We selected FA1 for modification by incorporating S.A., resulting in the fabrication of a composite hydrogel named FA1+S.A. FA1 was modified by adding 0.3%, 0.6%, and 0.9% S.A. to investigate the effects of these concentrations on the insulin release profile in vitro. At first, 2 mg of lyophilized peptide (FA1) was dissolved in 20 mm PB, pH 7.4. Simultaneously, a 2% stock solution of S.A. was prepared by dissolving it in the same buffer and then continuously agitated on the heater cum magnetic stirrer for ∼ 1 h. After that, the appropriate volume of 2% S.A. was mixed with the contents to attain the final concentration of S.A. in the 200 µL final volume as 0.3%. After resuspending the peptides in this solution, the powder was dissolved in 6N NaOH, and then 300 mM NaCl was added to modulate stiffness. The pH was adjusted to 7.4 again with 6N HCl, resulting in the formation of the composite hydrogel. The same process was followed to prepare the other composite hydrogels with S.A. of 0.6% and 0.9%.

### 4.4 Transmission Electron Microscopy (TEM)

TEM analysis was carried out to determine the hydrogel's morphology. The hydrogels (1:50 dilution in 20 mM PB, pH 7.4) were spotted onto a carbon‐coated Formvar grid (glow‐discharged) and incubated for 10 min. Next, the grids were washed with MQ water, then stained with an aqueous uranyl formate solution (2% w/v) for 1 min. Imaging was performed using JEOL Field Emission Gun‐transmission electron microscopy (JEM 2100 F, JEOL, Japan) at 200 kV with X25,000 magnification. Three independent biological experiments were carried out.

### 4.5 Scanning Electron Microscopy (SEM)

To perform SEM analysis, hydrogels were prepared (1:50 dilution), after which 10 µL of the samples were drop‐cast on a glass coverslip and allowed to dry at RT. After drying, the samples were platinum‐coated for 120 s at 10 mA. Micrographs were acquired in a JEOL JSM‐7600F (Peabody, MA, USA) at 10 kV with magnifications of X50,000 and X100,000. Three independent experiments were carried out, and the diameter of at least 50 fibrils was measured for each hydrogel using ImageJ v1.54 (NIH, Bethesda, USA). Three independent biological experiments were carried out.

### 4.6 Atomic Force Microscopy (AFM)

For carrying out AFM studies, 20 µL of vortexed hydrogel samples (1:50 dilution) were spotted on mica sheets (freshly cleaved) and incubated (10 min) at RT. They were subsequently dried overnight in vacuum desiccators. Image acquisition was done with a silicon nitride cantilever in tapping mode using AFM (Asylum Research, Santa Barbara, CA). Two independent biological experiments were performed, and five distinct areas were randomly scanned (scan rate of 1.5 Hz).

### 4.7 Cryo‐Scanning Electron Microscopy (Cryo‐SEM)

The hydrogel samples were placed onto the carbon tape over the metallic stub and then frozen in liquid nitrogen. Then they were inserted into the preparatory chamber. The hydrogel samples were freeze‐fractured with a cooled knife and then sublimed at ‐90°C for 10 min. At the end, the samples were platinum‐coated (sputter coating) for 45 s at 10 mA. Micrographs were acquired at X5000 magnification, using a JSM‐7600F SEM with PP3010T cryo‐unit (Quorum). The pore size of the hydrogels was calculated in ImageJ software from the acquired images. 5 randomly selected fields were employed to calculate at least 25 pores for the hydrogels. Three independent biological experiments were performed.

### 4.8 Rheological Measurements

A total of 300 µL of preformed hydrogel samples were placed between parallel plates (PP25 geometry). At 37°C, measurements of the dynamic oscillatory mode were carried out using a 0.2 mm gap size and a constant amplitude (0.05%). The frequency sweep analysis was conducted over an angular frequency (0.1–100 s^−1^) for a time period of 15 min. The thixotropicity was ascertained for the step‐strain rheology, using the same setup. After the gel was disrupted by applying a 100% strain, it was allowed to recover by lowering the strain to 0.05%. Over a period of three decades, the two moduli were calculated and plotted as functions of time. These measurements were performed in an Anton Paar rheometer (Graz, Austria). Two independent biological experiments were carried out.

### 4.9 Fourier Transform Infrared (FTIR) Spectroscopy

First, the KBr pellet was prepared by compressing the powder onto which 20 µL of the hydrogel samples were drop‐cast. 20 µL of 20 mM PB (pH 7.4) was drop‐cast onto another pellet to obtain the background spectra. The spectra were acquired in a Vertex 80 FTIR system (with a DTGS detector, Bruker, Leipzig, Germany). After an average of 32 scans, the spectra were acquired in the 1800–1500 cm^−1^ range at a resolution of 4 cm^−1^. All raw spectra were subjected to Fourier self‐deconvolution to resolve the amide I region (1600–1700 cm^−1^), and Opus‐65 software (Bruker, Leipzig, Germany) was employed to fit a Lorentzian curve. Three independent biological experiments were carried out for each test.

### 4.10 Thioflavin T (ThT)‐Binding Assay

For this assay, 3 µL of a 1 mm ThT dye solution (prepared in 10 mm Tris‐HCl buffer, pH 8.0, 0.01% sodium azide) was added to 200 µL of vortexed hydrogel, which was diluted 20‐fold in PB (20 mm, pH 7.4). The contents were mixed in the tube and then transferred to a quartz cuvette. Using an excitation (450 nm), an emission (460–500 nm), and a 5 nm slit width for excitation & emission, the fluorescence was measured right away in a Spectrofluorimeter (JASCO FP 8500, USA). 200 µL of PB was mixed with 3 µL of a 1 mm ThT dye solution for the ThT control experiment. The data were background‐corrected and plotted at the emission maximum (λmax = 480 nm). Three independent biological experiments were performed.

### 4.11 Congo Red (CR)‐Binding Assay

A total of 15 µL of CR dye (100 µm in PBS, pH 7.4, with 10% absolute ethanol) was added to 100 µL of hydrogel (10X diluted in PBS (20 mm, pH 7.4)) and then left for 5 min incubation in the dark. Using a UV spectrophotometer (JASCO V‐650, Tokyo, Japan), UV absorption curves were recorded from 300–700 nm. The dye was incubated with the PB (20 mm, pH 7.4) to conduct the CR control experiment. Three independent biological experiments were carried out.

### 4.12 Study of Hydrogel Swelling

A total of 300 µL of the hydrogel was placed in a 35‐mm cell culture dish (Costar, Corning, NY, USA). After gelation, the initial weight of the dish with the drop‐cast hydrogel was determined and defined as W_i_. Then, 2 mL of PBS (pH 7.4) was added, and the dish was incubated at 37°C for 24 h. After 1 day, the dish was taken out to determine the final weight (W_f_) after excess water was removed from its surface. The swelling ratio (%) was determined according to the following formula: [W_f_—W_i_) / W_i_] × 100%

Three independent biological experiments were carried out.

### 4.13 Evaluation of In Vitro Cytocompatibility of the Hydrogels

The MTT (3‐[4,5‐dimethylthiazol‐2‐yl]‐2,5‐diphenyl tetrazolium bromide) assay was performed using an NIH 3T3 cell line to assess the cytocompatibility of the hydrogels over 24 h. For this, in 96‐well plates, 5 µL of hydrogel was used to make a bed and air‐dried, after which NIH 3T3 (∼10,000) cells in 100 µL complete DMEM were seeded and incubated (24 h). Untreated and Triton‐X (0.2%) treated cells were used as positive and negative controls, respectively. After 24 h, 10 µL of MTT [5 mg/mL] was added to each well. Following this, 100 µL (per well) of a 50% DMF‐20% SDS solution was added after 4 h of MTT addition, and the samples were incubated overnight. In a VANTAstar microplate reader (BMG LABTECH, Germany), background scattering at 690 nm and absorption at 560 nm were both measured. For assessing cell viability, background scattering was subtracted from the 560 nm absorbance. To compute the cell viability percentage, the absorbance of each test sample was normalized to that of the corresponding control and expressed as a percentage. Three independent biological experiments were carried out.

### 4.14 Evaluation of Hemocompatibility of the Hydrogels

The hydrogels were assessed for their hemolytic toxicity. Fresh blood was collected from male adult Wistar rats, 6–8 weeks old, weighing around 150–200 g. The blood was first centrifuged at 4°C and 1100 r.p.m. for 15 min to settle the RBCs. PBS and MQ water were considered negative and positive controls, respectively. Three concentrations of the amyloid hydrogel (vis, 5 mg/mL, 10 mg/mL, and 15 mg/mL) and composite hydrogels with different S.A. concentrations were assessed in the test. 50 µL of RBCs (per tube) were slowly added and mixed, after which the tubes were incubated at 37°C, 5% CO_2_, 95% humidity for 4 h. Then, the samples were centrifuged at 3000 r.p.m. at 4°C for 5 min. The supernatant (50 uL) was used to measure 540 nm absorbance using a VANTAstar microplate reader. The percent hemolysis was computed by the equation given below:

$$\mathrm{Hemolysis}\;\left(\%\right)=\frac{\text{Sample absorbance}\;-\;\text{negative control}}{\text{Positive control}\;-\;\text{negative control}\;}\times 100$$

Three independent biological experiments were carried out.

### 4.15 Fluorescent Labeling of Insulin

The FITC labeling of insulin was carried out according to the manufacturer's protocol (Thermo Fisher Scientific, USA). Briefly, an excess (4X molar) of FITC (dissolved in DMSO) was added to the insulin solution. For FITC labeling, it was incubated on a magnetic stirrer (at 4°C for 6 h) in the dark with low‐speed rotation. The excess dye was removed via dialysis through a 1‐kDa molecular‐weight cutoff (MWCO) membrane in 10 mm PBS (pH 7.4) at 4°C for 48 h. The labeled insulin was then lyophilized, and its concentration was determined by redissolving the dry weight in 10 mm PBS (pH 7.4) according to the manufacturer's protocol.

### 4.16 Preparation of FITC‐Labeled Insulin‐Loaded Amyloid Hydrogels

FITC‐labeled insulin was dissolved in PBS (pH 7.4) and made to dissolve completely using 6N HCl at 150 mg/mL final concentration. Then, appropriate concentrations of insulin were encapsulated in the hydrogels by simply tapping them and keeping them for 15 min to prepare FITC‐insulin‐loaded hydrogels.

### 4.17 Insulin Loading Determination

The encapsulation efficiency (EE%) of the protein drug inside the drug depots was determined. For this purpose, the hydrogel was encapsulated with 4 different concentrations of FITC‐labeled insulin, viz., 1.25 mg/ mL, 2.5 mg/ mL, 5 mg/ mL, and 7.5 mg/ mL. Following encapsulation, the hydrogels were left for 15 min to ensure gelation, followed by layering 1 mL of PBS (pH 7.4) over the FITC‐insulin‐encapsulated hydrogels and immediately aspirating to quantify the FITC‐insulin concentration in the supernatant in a Spectrofluorimeter (JASCO FP 8500, USA). The FITC‐insulin concentration encapsulated within the hydrogel was ascertained by subtracting the concentration of released FITC‐insulin in the supernatant from the total concentration of FITC‐labeled insulin loaded using the following formula:

$$\begin{array}{cc} & \mathrm{EE}\left(\%\right)\\ & =\left[1-\left(\mathrm{Unencapsulated}\;\mathrm{FITC}-\mathrm{insulin}/\mathrm{Total}\;\mathrm{FITC}\right.\right.\\ & \left.\left.-\mathrm{insulin}\;\mathrm{encapsulated}\right)\right]\times 100\% \end{array}$$

Three independent biological experiments were carried out

### 4.18 In Vitro Drug Release Assay

In vitro release assays were conducted to assess the hydrogel delivery system's ability to release insulin effectively. The hydrogels were encapsulated with the required concentrations of FITC‐ insulin 15 min prior to the start of the release assay to ensure proper gelation. Following this, 1 mL of prewarmed 10 mm PBS (pH 7.4) was layered over 300 µL of this hydrogel, and the mixture was incubated at RT. 100 µL of PBS was withdrawn from the tubes at predetermined time points (1 to 90 h for amyloid hydrogels and 1 to 102 h for composite hydrogels) and replaced periodically with fresh buffer (100 µL) to sustain sink conditions. The FITC‐insulin concentration released from the system was determined using the Spectrofluorimeter (JASCO FP 8500, USA). The cumulative FITC‐insulin concentration released was determined using the FITC‐insulin standard curve in combination with the following formula:

Cumulative FITC‐insulin released (%) = $\frac{At}{Ai}$ × 100, where At is the concentration of FITC‐insulin released at time t, and Ai is the total concentration of FITC‐insulin loaded. The drug‐release data for peptide‐based amyloid hydrogels and composite hydrogels were fitted to zero‐order, first‐order, Higuchi, and Korsmeyer‐Peppas models to assess the goodness of fit and the drug‐release mechanism. Additionally, for composite hydrogels, the release data were fitted to the zero‐order kinetic model to calculate the release rate, and the resulting release rate was then analyzed for correlation with the FRAP‐derived diffusion coefficient of insulin. Three independent biological experiments were carried out.

### 4.19 Fluorescence Recovery After Photobleaching (FRAP) of FITC‐Insulin Encapsulated in Hydrogels

FITC‐insulin was encapsulated in the hydrogels and then used for FRAP studies, in which the fluorescent area was bleached, and recovery was monitored as per the established protocol on an Olympus FV3000LSM (with an in‐built FRAP module) [92, 93]. Additionally, two other regions of interest (ROI) with similar diameters were used for passive bleaching and background correction. Photobleaching of the ROI at its center was achieved with a 488‐nm DPSS laser. Images were acquired for 100 s with a 512 × 512 frame and 16‐bit depth. The data were bleach‐corrected and normalized in cellSens software and fitted in OriginPro 2021 v9.8.0 (Origin‐Lab, USA) employing an exponential growth function to determine the half‐time (t_1/2_) and diffusion coefficients according to the previously published protocol [92, 93]. Three independent biological experiments were carried out.

### 4.20 Ion‐Pair High‐Performance Liquid Chromatography (IP‐HPLC)

We used IP‐HPLC with hexane sulfonate as an ion‐pairing reagent [94] to assess the chemical integrity of the released insulin samples, with UV detection at 195 nm. Insulin solution (freshly prepared) was used as a control. IP‐HPLC was carried out on a Shimadzu LC20‐AR instrument (with a Shimadzu C18 column: 250 mm long × 4.6 mm diameter, 5 µm particle size). The mobile phase was an aqueous solution of 50 mm KH_2_PO_4_ and 7.2 mm sodium hexanesulfonate, pH 2.5, used isocratically at a 1 mL/min flow rate. The chromatograms display absorbance (in mV units) versus retention time and were plotted using OriginPro 2021 v9.8.0 (OriginLab, USA). Three independent biological experiments were carried out.

### 4.21 MALDI‐TOF Mass Spectrometry

MALDI‐TOF MS was used to assess the structural integrity of the hydrogel‐released insulin samples. The investigation was performed in an Autoflex Speed MALDI‐TOF mass spectrometer (Bruker Daltonics Inc., Billerica, Massachusetts). Here, the samples were added to Sinapinic acid (trans‐3,5‐dimethoxy‐4‐hydroxycinnamic acid) at a 1:1 ratio. Then, it was spotted immediately onto the target plate and then dried at RT for 10 to 15 min. Insulin solution (freshly prepared) was used as a standard. Data acquisition was performed using Flex control software, followed by data analysis with Flex analysis, in accordance with the manufacturer's instructions. The data were plotted using OriginPro 2021 v9.8.0 (OriginLab, USA). Three independent biological experiments were carried out.

### 4.22 Tris‐Tricine Sodium Dodecyl Sulfate (SDS)‐PAGE

Tris‐Tricine SDS‐PAGE was performed under non‐reducing conditions to examine insulin's structural integrity and potential aggregation after loading and release activities. Before loading the samples into the wells, the released samples at various time points were mixed with a non‐reducing 1X loading buffer (250 mm Tris‐HCl, pH 6.8, 10% SDS, 30% glycerol and 0.02% bromophenol blue), and then resolved through the polyacrylamide gel consisting of resolving (15%), spacer (10%) and stacking (4%) gel, first at 50 V for stacking the proteins, and then at 100 V for ∼5‐6 h. Insulin solution (freshly prepared) was used as a standard, and the gels were run in two buffer systems: anode buffer (pH 8.9; 30 mm Tris‐HCl) and cathode buffer (pH 8.25; 100 mm Tris‐HCl, 100 mm Tricine, and 3.5 mm SDS). Proteins were stained with Coomassie Brilliant Blue R 250. The molecular weights of the detected bands were compared with those of freshly prepared insulin and the pre‐stained protein marker. Two independent biological sets of experiments were performed.

### 4.23 Circular Dichroism (CD) Spectroscopy

The samples were taken in a thoroughly cleaned quartz microcuvette (Hellma cells, UA) with a 0.1 cm path length, and spectra were acquired at 25°C using a CD instrument (JASCO‐1500) in the 198–260 nm wavelength range. Three accumulations were acquired for each spectrum, and the average was used. Following that, the raw data were smoothed and buffer‐subtracted. Spectra Manager R2 software (Jasco) was used for spectral analysis. The data were plotted using KaleidaGraph v4.03 software. Three independent biological experiments were carried out.

### 4.24 Protein Expression and Purification, and Aggregation Kinetics Study

Expression of WT α‐Syn was executed in *Escherichia coli* BL21(DE3) cells according to the established protocols of Volles et al., with some modifications [95]. A low molecular weight solution and preformed fibrils of WT α‐Syn were prepared in 20 mM PB containing 0.01% sodium azide at pH 7.4 according to the protocol described by Ghosh et al. [96]. Preformed fibril seeds were prepared using a probe sonicator (Sonics & Materials Inc., Newtown, CT, USA), operated at 20% amplitude for 3 min (3 pulses on, 1 pulse off). Then, 1% and 1.5% (v/v) seeds of both preformed WT α‐Syn fibril and FA1 hydrogel fibril were added to 50 µL of WT α‐Syn solution (300 µm). The fibrillation was monitored by measuring the ThT fluorescence intensity in a plate reader. As controls, seeds‐only and WT α‐Syn‐only were incubated under identical experimental conditions.

The hIAPP peptide was a kind gift from Prof. Anirban Bhunia of the Bose Institute, Kolkata. It was initially dissolved in HFIP at 1 mg/mL and aliquoted into separate vials, each containing 200 µL. The aliquots were lyophilized for 48 h and then stored at −20°C until use. To prepare the peptide solution for the aggregation kinetics setup, the lyophilized peptide was reconstituted in chilled 0.1N HCl, 20 mm PB, pH 7.4 (containing 50 mm NaCl) and AMQ, then thoroughly mixed on ice. To prepare IAPP seeds, the reconstituted peptides were incubated at 37°C (200 rpm) for at least 24 h with agitation. Then, 1% and 1.5% (v/v) seeds of both preformed IAPP fibril and FA1 hydrogel fibril were added to 50 µL of IAPP solution (20 µm). The fibrillation was monitored at 37°C by measuring ThT fluorescence intensity in a VANTAstar microplate reader (shaking for 1 min at 500 rpm before each reading cycle). As controls, seeds‐only and IAPP‐only were incubated under identical experimental conditions.

The acquired data (at 480 nm) were fitted to a sigmoidal curve using GraphPad Prism v9.5.1. Three independent biological experiments were conducted.

### 4.25 In Vivo Studies

All animal experiments were approved by the Institutional Animal Ethics Committee of the Institute of Chemical Technology, Mumbai, India (Approval Certificate No.: ICT/IAEC/2022/P18). Healthy male Wistar rats, 6–8 weeks old, weighing ∼150‐200 g, were procured from the National Institute of Biosciences, Pune, India, and acclimatized for 7 days. All animals were maintained *in‐house* under standard conditions (50% humidity, 22 ± 2°C, and a 12 h dark/light cycle) with ad libitum access to food and water.

Type 1 diabetes was induced by a single i.p. injection of STZ (40 mg/kg b.w.) freshly prepared in cold citrate buffer (0.1 m, pH 4.5). To avoid acute hypoglycemia, they received a 10% (w/v) glucose solution in drinking water immediately after STZ administration. BGLs were measured 3 days later using a glucometer, and rats with BGL >250 mg/dL were considered diabetic and used for subsequent experiments. For therapeutic efficacy studies, diabetic rats were randomly assigned to receive s.c. injections of PBS, FA1 hydrogel, insulin‐loaded FA1 hydrogel, free insulin, and Lantus. BGLs were monitored at predetermined time points, and serum insulin was determined using a human ELISA kit. The bioactivity of hydrogel‐released insulin during storage and physiological release was similarly evaluated by s.c. administration of the released insulin samples into diabetic rats, followed by BGL measurements.

An IPGTT was subsequently performed in glucose‐treated normal and diabetic rats (*n* = 6) following a 6 h fast. Animals received an i.p. glucose challenge (1 g/kg b.w.), followed by s.c. administration of PBS, free insulin, and FA1+insulin formulation. BGLs were monitored at the indicated time points to evaluate glucose clearance and therapeutic efficacy.

### 4.26 Long‐Term In Vivo Biosafety Evaluation of Functional Amyloid Hydrogel

To assess the biological safety of our formulation in vivo, 6–8‐week‐old male Wistar rats (*n* = 5 per group) were treated for 15 days with a repeated dose every 2.5 days. The first group consisted of normal, untreated, healthy rats that were injected subcutaneously with PBS (pH 7.4), while the remaining 3 groups were diabetic and were used to evaluate probable toxicity, with s.c. injection of PBS (pH 7.4), FA1+insulin, and FA1 alone. Body weights and water intake of the rats in the four groups mentioned above were continuously monitored on days 1, 7, and 15. For serum biochemistry profiling, the rats were euthanized on day 15, and serum was analyzed for potential changes in biochemical and lipid parameters (including complete blood count, hemoglobin, albumin, bilirubin, creatinine, ALT, AST, ALP, cholesterol, triglycerides, HDL, and LDL). Moreover, two immunological markers in the serum, namely Interleukin‐1β (IL‐1β) and tumor necrosis factor‐α (TNF‐α), were measured on the last day using an ELISA kit (Krishgen Biosystems, Mumbai, India) to assess any possible immunoreactivity in the body resulting from the formulation's introduction. Also, tissue samples from major organs were obtained for H/E staining, and their gross histology and immune cell infiltration were examined to assess potential tissue lesions. All essential organs were extracted and fixed in 10% v/v formalin (Merck, Germany). Subsequently, the specimen was paraffin‐wax‐embedded, and microtome‐cut sections (5 µm thick) were prepared and examined under a DMi8 microscope (Leica Microsystems, Germany) equipped with a DMC2900 DOC camera (Leica Microsystems, Germany) for assessing pathological alterations. Moreover, the rat skins were stained with MT to assess potential collagen deposition resulting from the injection. The density of collagen deposition in each group was also calculated using ImageJ v1.54 (NIH, Bethesda, USA) software.

### 4.27 Statistical Analysis

Data are presented as mean ± S.E.M. from three independent biological experiments unless otherwise indicated. Statistical analyses were performed using GraphPad Prism v9.5.1. Comparisons between two groups were conducted using two‐tailed paired or unpaired t‐tests with Welch's correction, as appropriate. Comparisons among multiple groups were performed using one‐way analysis of variance (ANOVA) followed by Dunnett's or Tukey's multiple‐comparisons test, as appropriate. The statistical significance was calculated at the 95% confidence level, with *p*‐values of < 0.0001, < 0.0002, < 0.0021, < 0.0332, and > 0.1234, indicated by (^****^), (^***^), (^**^), (^*^), and (ns), respectively.

## Author Contributions

R.S. designed and performed the experiments, analyzed and curated the data, validated the findings, and wrote the original manuscript. N.M.A.C. and S.M. performed experiments, contributed to data analysis and curation, and reviewed and edited the manuscript. S.S. provided resources, supervised the study, and reviewed the manuscript. S.K.M. conceived the study, secured funding, supervised the project, provided resources, and reviewed and edited the manuscript. All authors read and approved the final manuscript.

## Conflicts of Interest

The authors declare no conflicts of interest.

## Supporting information




**Supporting File**: advs78568‐sup‐0001‐SuppMat.pdf.

## Data Availability

The data that support the findings of this study are available from the corresponding author upon reasonable request.
