# Supplementary material for: Injectable Peptide‐Based Functional Amyloid Hydrogels as Tunable Depots for Sustained Drug Delivery
Source: Adv Sci (Weinh). 2026 Oct 29:e78568. Online ahead of print. doi: 10.1002/advs.78568 (PMC13720263; doi:10.1002/advs.78568)
Supplement: Supplementary file 1 [file ADVS-9999-e78568-s001.pdf]

SUPPORTING INFORMATION

Injectable Peptide-Based Functional Amyloid Hydrogels as Tunable Depots for Sustained Drug Delivery

Ranjit Shaw[1], Niyamat M. A. Chimthanawala[3], Shouvik Manna[1], Sadhana Sathaye[3], Samir K. Maji[1,2*]

[1]Department of Biosciences and Bioengineering, Indian Institute of Technology Bombay, Powai, Mumbai, 400076, India

[2]Sunita Sanghi Center of Aging and Neurodegenerative Diseases (SCAN), Indian Institute of Technology Bombay, Powai, Mumbai, 400076, India

[3]Department of Pharmaceutical Sciences and Technology, Institute of Chemical Technology, Mumbai, 400019, India

*Correspondence: Prof. Samir K. Maji, Department of Biosciences and Bioengineering, Indian Institute of Technology Bombay, Powai, Mumbai, 400076, India.

Tel: + (91-22) 2576-7774, Fax: + (91-22) 2572 3480.

Email: samirmaji@iitb.ac.in

Contents

S. No.		Page Nos.
1.	Supporting Figures (1-15)	3-17
2.	Supporting Tables (1-2)	18
3.	References	19

Supporting Figures

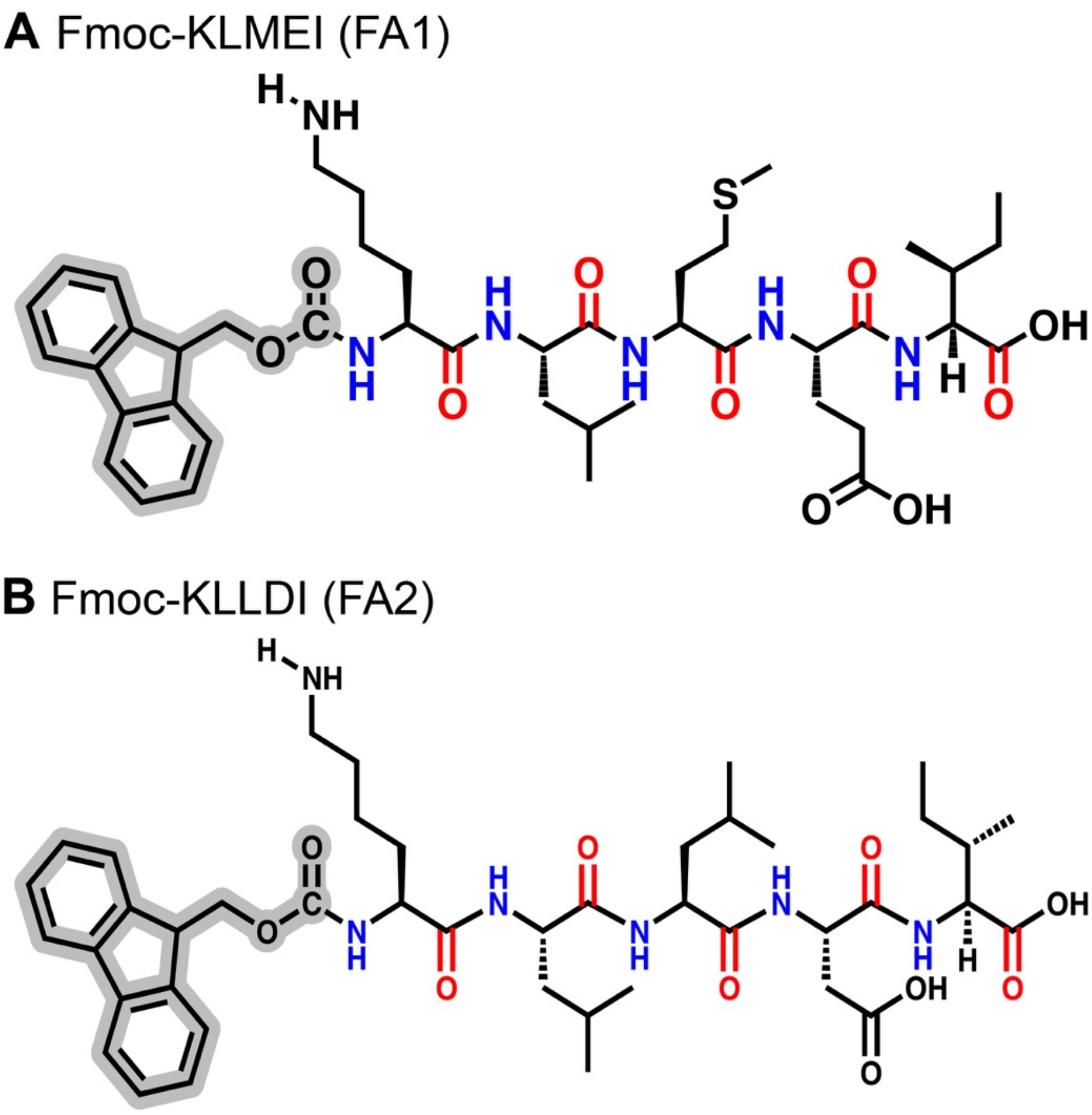

FIGURE S1 | Chemical structure of the pentapeptides used for the fabrication of functional amyloid hydrogels. The Fmoc-protected pentapeptides employed for hydrogel formation are shown. Upon pH-triggered self-assembly, these peptides form supramolecular amyloid fibrillar networks that serve as injectable hydrogels for sustained protein delivery. The fluorenyl methoxycarbonyl (Fmoc) group is highlighted in gray. These structures were drawn using ChemDraw v22, and the figure was prepared and assembled in Adobe Illustrator v27.0.

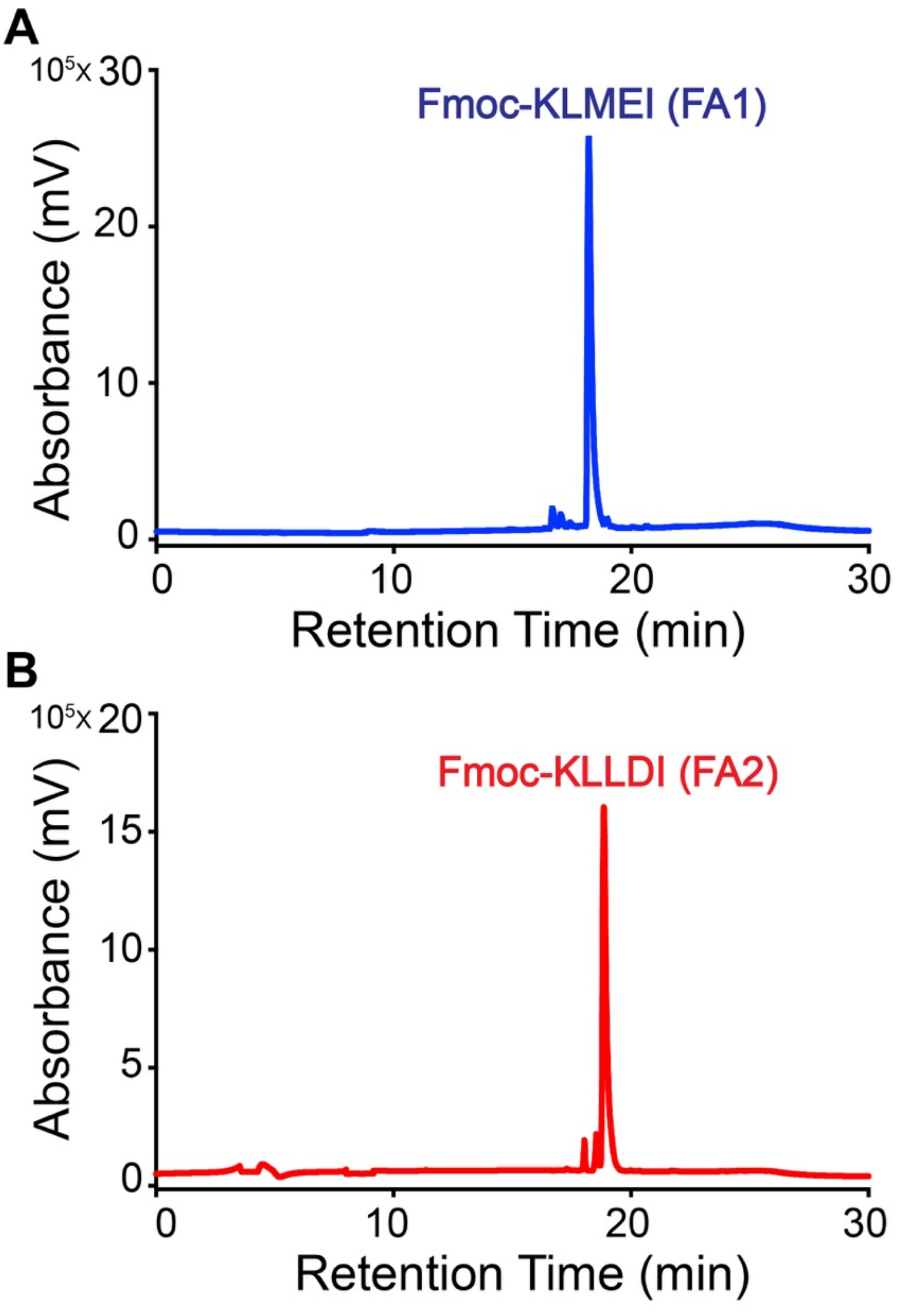

FIGURE S2 | Analytical HPLC characterization of the synthesized peptides. Representative analytical HPLC chromatograms recorded at 254 nm showing a major peak for each peptide with a retention time of approximately 18-20 min, indicating high peptide purity. Data were plotted and analyzed using GraphPad Prism v9.5.1, and the figure was prepared and assembled using Adobe Illustrator 2023 v27.0.

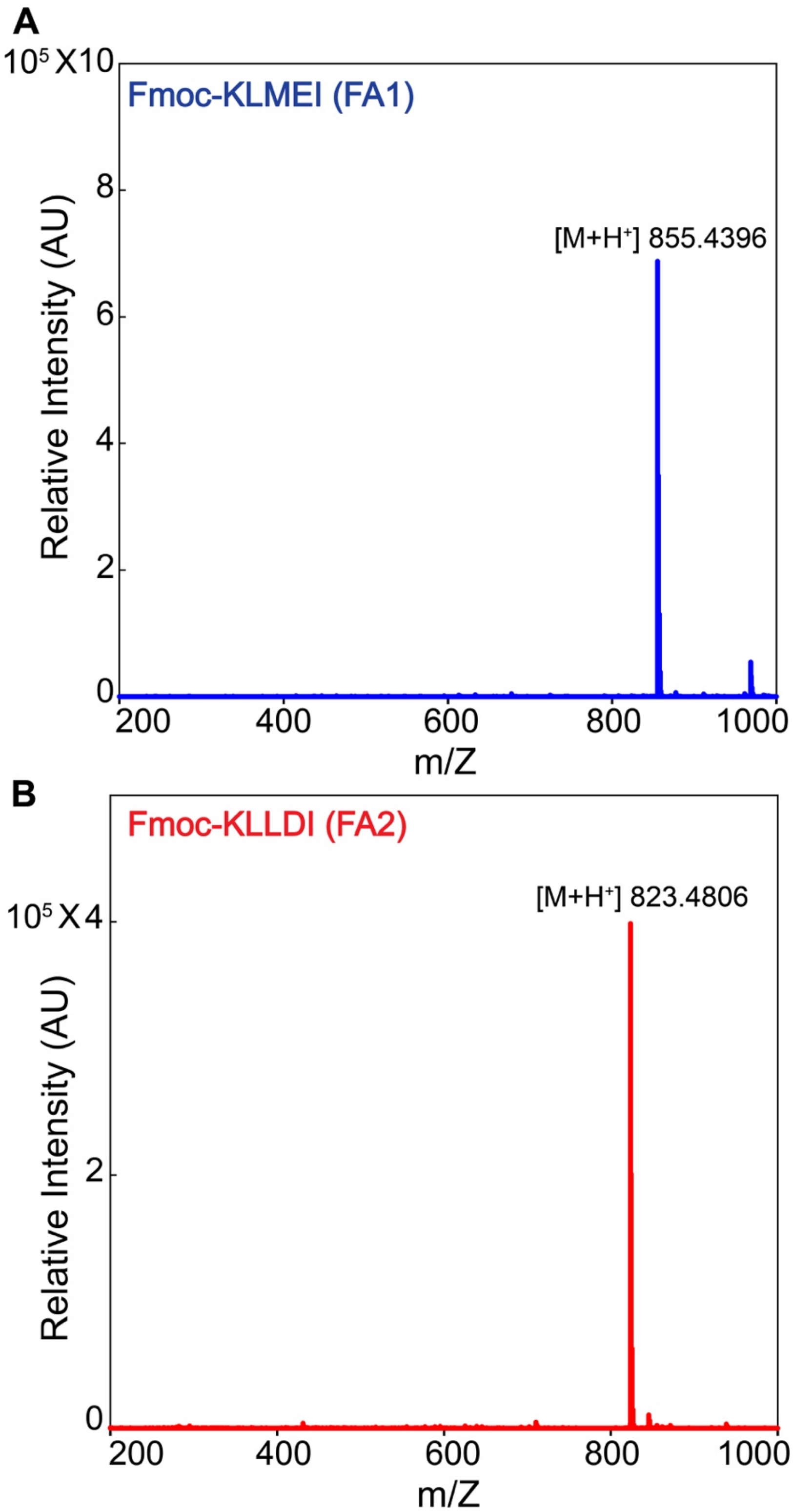

FIGURE S3 | ESI-MS characterization of the synthesized pentapeptides. It shows the ESI-MS spectra of both peptides used to synthesize the amyloid hydrogels. The molecular weights of the peptide sequences are represented by the predominant molecular ion peaks in the peptides' ESI-MS spectra, thereby confirming the identity of the peptide sequences used for hydrogel fabrication. Data were plotted and analyzed using GraphPad Prism v9.5.1, and the figure was prepared and assembled using Adobe Illustrator 2023 v27.0.

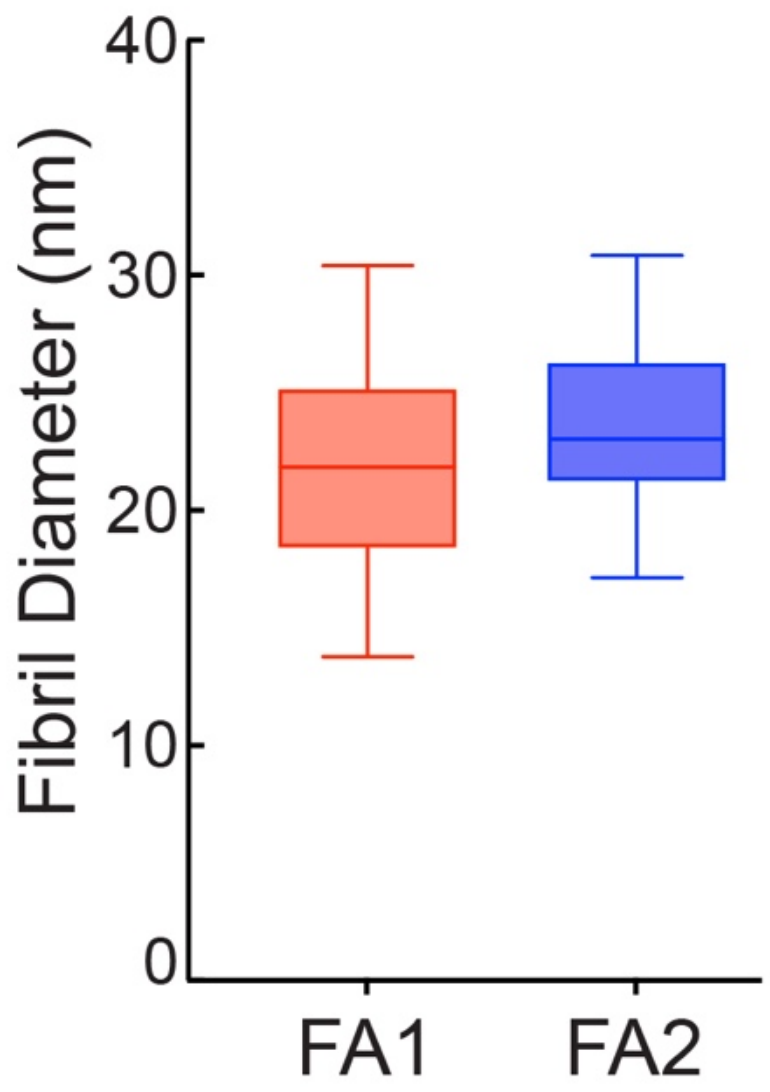

FIGURE S4 | Quantitative analysis of fibril diameter of the peptide-based functional amyloid hydrogels. Box plot showing the distribution of fibril diameters measured from at least 50 fibrils per hydrogel. The quantified fibrils exhibit diameters predominantly in the range of ~15-25 nm, consistent with the characteristic dimensions of peptide-derived amyloid hydrogels. Data were plotted and analyzed using GraphPad Prism v9.5.1, and the figure was prepared and assembled using Adobe Illustrator 2023 v27.0.

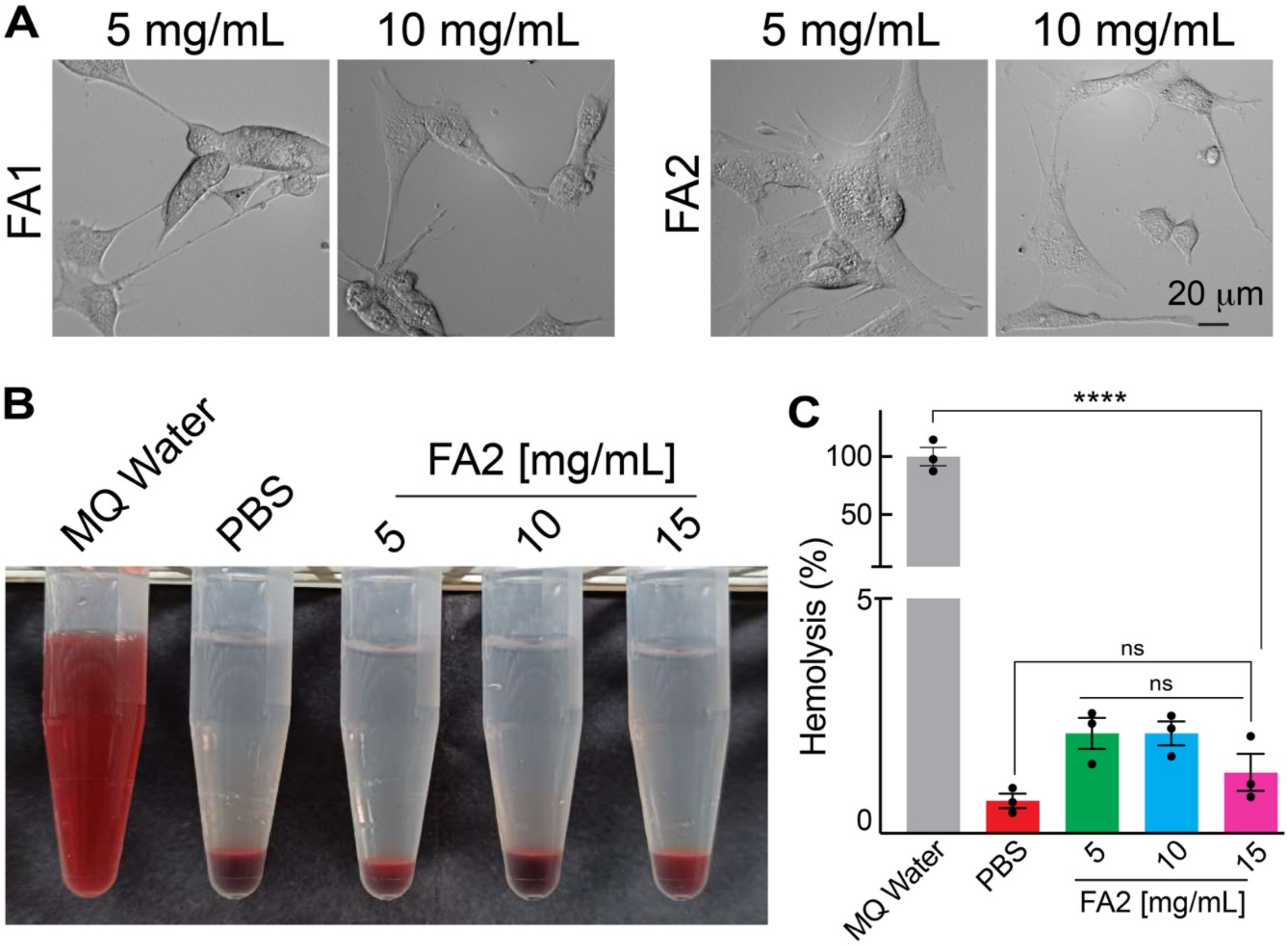

FIGURE S5 | Biocompatibility assay of the peptide-based amyloid hydrogels. (A) Bright-field representative images of the NIH 3T3 cells grown on different concentrations of the amyloid hydrogel-coated well plates. (B) Digital representative images of the corresponding hemocompatibility solutions of the hydrogel (FA2) at three different concentrations [5, 10, and 15 mg/mL] with erythrocytes, and corresponding quantification of percentage hemolysis under different conditions (C), showing that FA2 didn't induce any significant hemolysis. An intact RBC pellet was observed beneath the clear supernatant of the PBS and FA2 hydrogel, whereas the MQ-treated group showed a bright red supernatant without a pellet, confirming complete hemolysis. For C, the data represent the mean ± S.E.M. (n=3), and the statistical significance was determined using a one-way ANOVA, followed by a Tukey's multiple-comparison test [$^{****}p < 0.0001$, and $^{ns}p > 0.1234$ with a 95% confidence level]. Data were plotted and analyzed using GraphPad Prism v9.5.1 and ImageJ v1.54 (NIH, Bethesda, USA), as applicable, and figures were prepared and assembled using Adobe Illustrator 2023 v27.0.

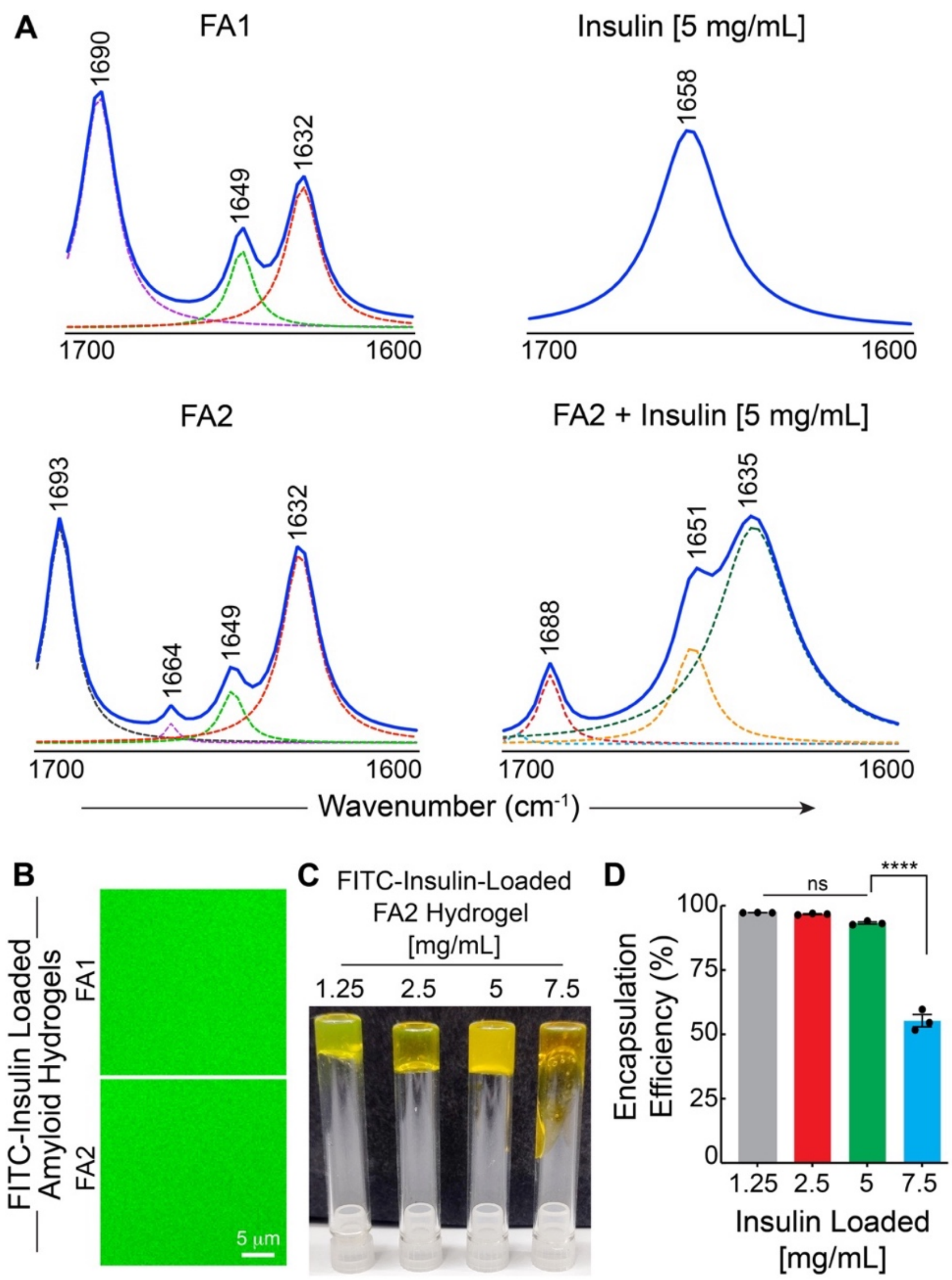

FIGURE S6 | Functional characterization of insulin-loaded amyloid hydrogels. (A) FTIR spectra of FA1, FA2, only insulin, and insulin-encapsulated FA2 hydrogel, demonstrating the presence of encapsulated insulin, as indicated by the presence of an alpha-helical secondary structure. (B) Representative confocal microscopy image of FITC-insulin-loaded amyloid hydrogel, showing uniform insulin encapsulation within the hydrogel matrix. (C) Digital representative image of the hydrogel tubes containing different concentrations of FITC-insulin, implying that FA2 can encapsulate ~95% insulin up to 5 mg/mL concentration of FITC-insulin, after which the gelation property of the hydrogel gets disturbed and leads to less encapsulation efficiency, as shown in the bar graph (D). For D, the data represent the mean ± S.E.M. (n=3), and the statistical significance was determined using a one-way ANOVA, followed by a Dunnett's multiple-comparison test [****$p < 0.0001$, and $^{ns}p > 0.1234$ with a 95% confidence level]. Data were plotted and analyzed using GraphPad Prism v9.5.1 and ImageJ v1.54 (NIH, Bethesda, USA), as applicable, and figures were prepared and assembled using Adobe Illustrator 2023 v27.0.

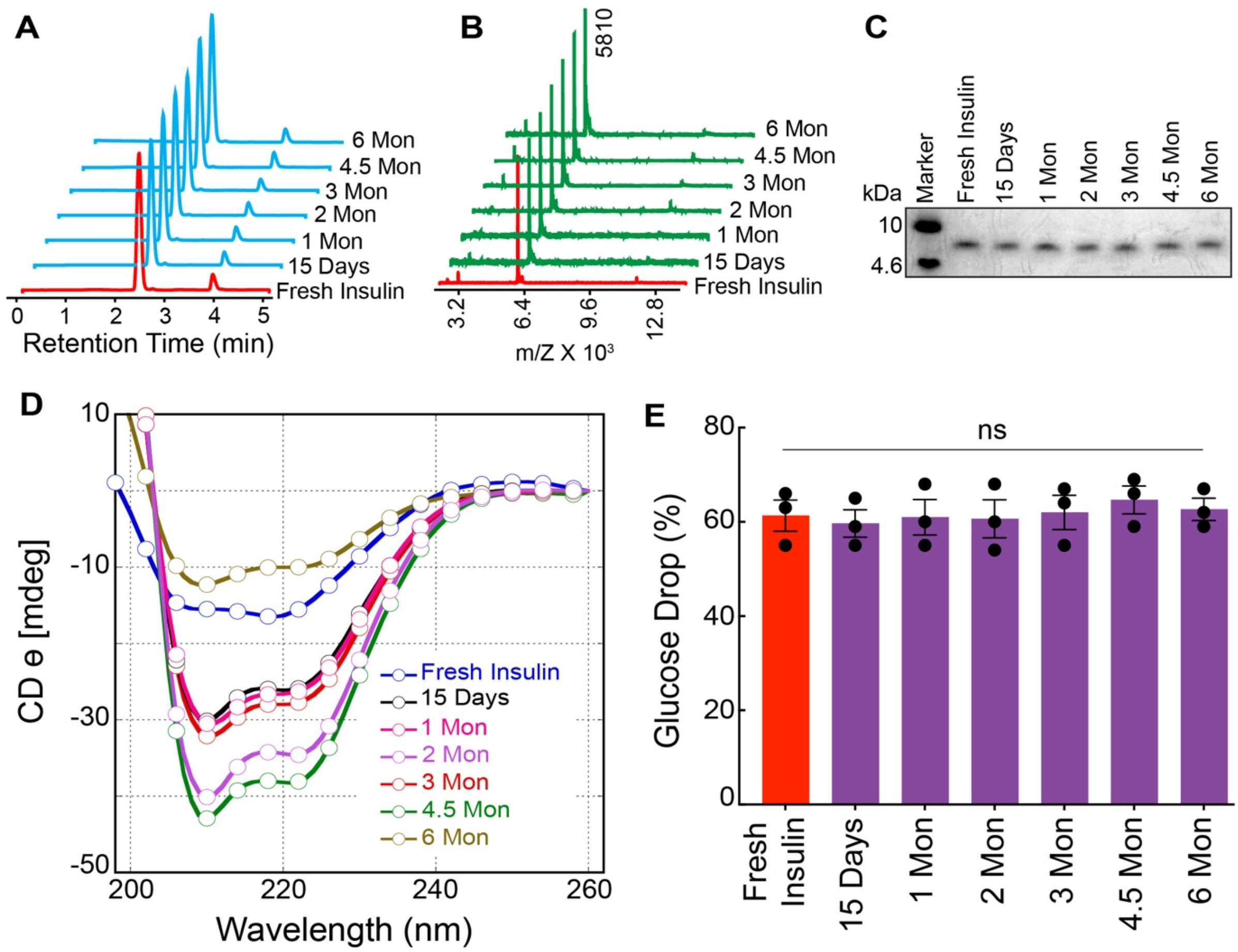

FIGURE S7 | Stability assay of insulin encapsulated within the FA2 amyloid hydrogel during storage at 4 °C for up to 6 months. (A) IP-HPLC chromatograms of insulin released from FA2 amyloid hydrogel at the indicated time points. (B) MALDI-TOF mass spectra confirming preservation of the molecular mass of the released insulin. (C)Tris-tricine SDS-PAGE analysis under non-reducing conditions, demonstrating retention of insulin integrity, as revealed by the presence of a single electrophoretic band between 4.6 and 10 kDa. (D) Far-UV CD Spectra showing preservation of the native secondary structure of released insulin from the hydrogel at different time points. (E) *In vivo* functional assay in STZ-induced Type 1 diabetic rats exhibiting that insulin released from FA2 amyloid hydrogel remains biologically active, as evidenced by its glucose-lowering effect. For E, the data represent the mean ± S.E.M. (n=3), and the statistical significance was determined using a one-way ANOVA, followed by a Dunnett's multiple-comparison test [$^{ns}p > 0.1234$ with a 95% confidence level]. Data were plotted and analyzed using GraphPad Prism v9.5.1, OriginPro 2021 v9.8.0, KaleidaGraph v4.03, and ImageJ v1.54 (NIH, Bethesda, USA), as applicable, and figures were prepared and assembled using Adobe Illustrator 2023 v27.0.

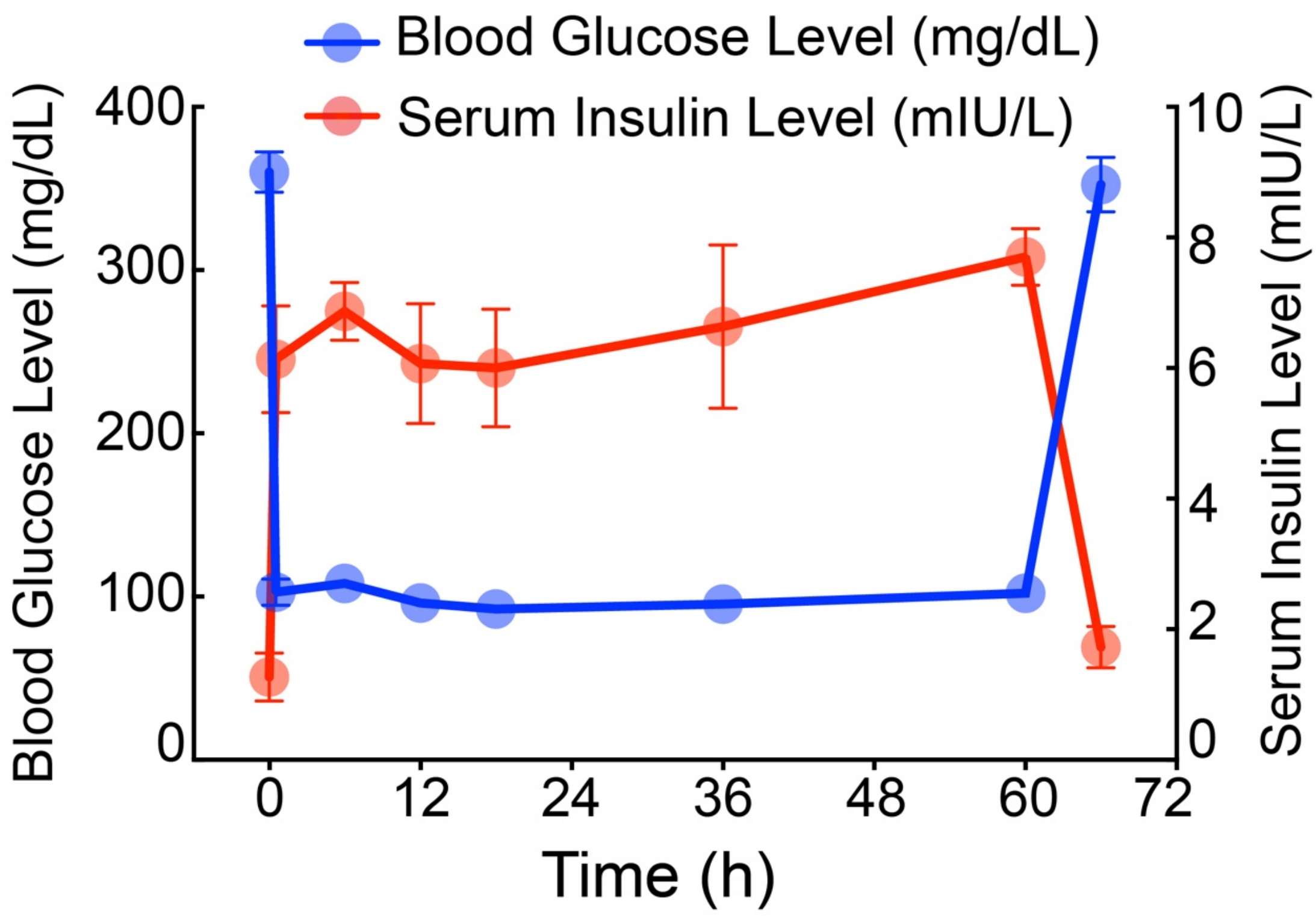

FIGURE S8 | Dual-axis representation of serum insulin concentration and blood glucose levels over time, following treatment, illustrating an inverse temporal relationship between the circulating insulin and glycemic regulation. The left y-axis shows blood glucose levels (mg/dL), and the right y-axis shows serum insulin concentration (mIU/L), highlighting the sustained insulin release and the corresponding prolonged glucose-lowering effect of the insulin-loaded amyloid hydrogel formulation. Data were plotted and analyzed using GraphPad Prism v9.5.1.

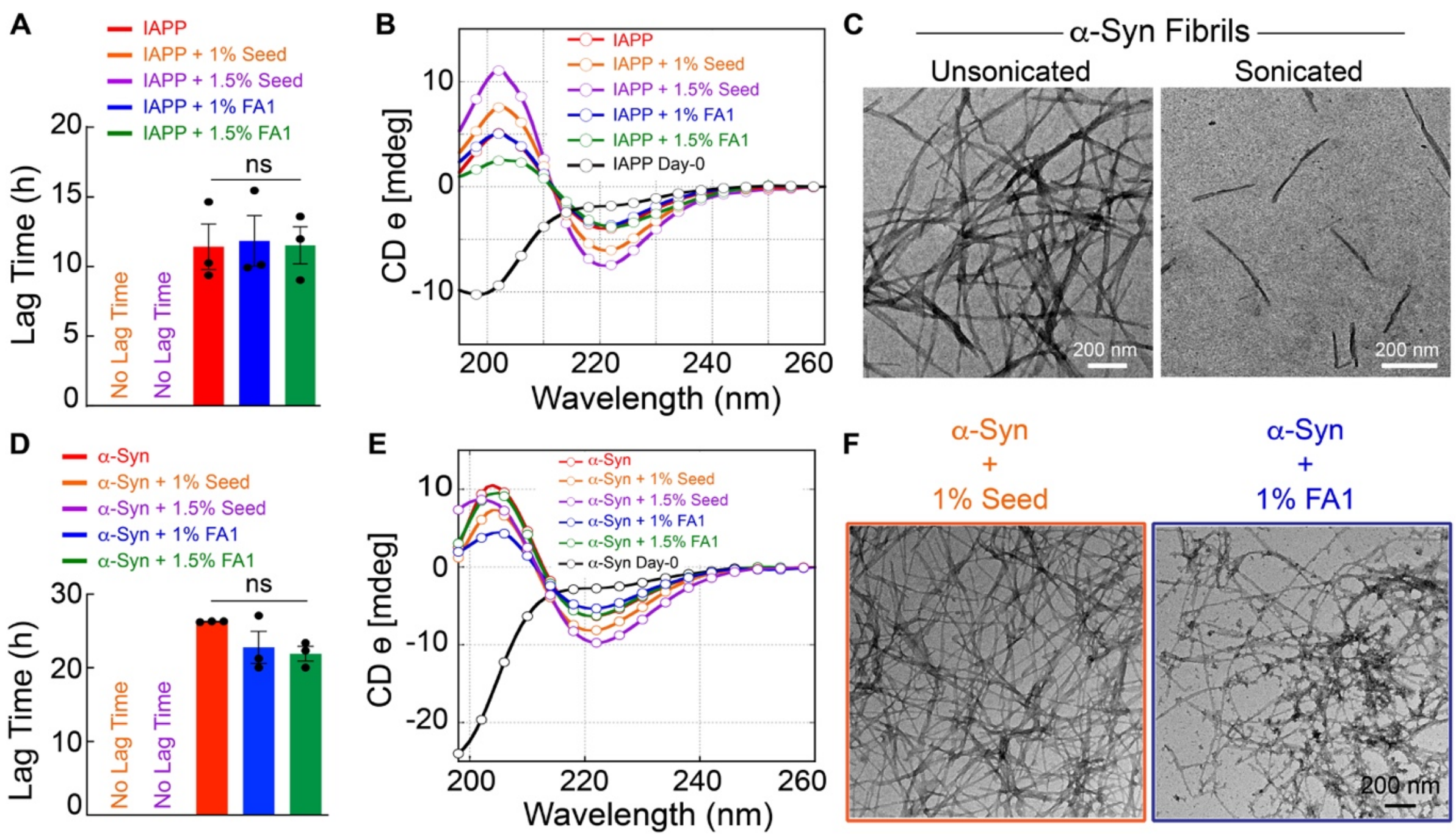

FIGURE S9 | Evaluation of heterologous cross-seeding potential of the FA1 functional amyloid hydrogel towards IAPP and α-Syn aggregation. (A) Lag times derived from the cross-seeding ThT fluorescence kinetics of IAPP incubated with varying concentrations of pre-formed IAPP seeds and FA1 amyloid hydrogel fibrils. It shows that IAPP pre-formed fibrils accelerated IAPP aggregation with no detectable lag time; however, FA1 fibrils did not significantly alter the lag phase, indicating the absence of detectable heterologous cross-seeding activity. (B) Far-UV CD spectra of IAPP showing random coil (unstructured) immediately after soluble protein preparation, but converted to β-sheet either alone or in the presence of preformed seeds. (C) Representative TEM images of the WT α-Syn matured (unsonicated) fibrils and respective sonicated pre-formed fibril seeds used as the positive control for homologous seeding experiments. (D) Lag times derived from the cross-seeding ThT fluorescence kinetics of α-Syn incubated with varying concentrations of pre-formed α-Syn seeds and FA1 amyloid hydrogel fibrils. While α-Syn pre-formed fibrils accelerated WT α-Syn aggregation in a concentration-dependent manner with no detectable lag time, FA1 fibrils did not significantly alter the lag phase, indicating the absence of detectable heterologous cross-seeding activity. (E) Far-UV CD spectra of WT α-Syn showing random coil (unstructured) immediately after soluble protein preparation, but converted to β-sheet either alone or in the presence of preformed seeds. The data indicate that amyloid hydrogels neither seeded the α-Syn aggregation nor delayed aggregation and the random coil-to-β-sheet transition. (F) Representative TEM images of the reaction products following heterologous seeding with 1% pre-formed seeds and 1% FA1 hydrogel fibrils, corroborating the biophysical analyses. The scale bar is 200 nm. For A and D, the data represent the mean ± S.E.M. (n=3), and the statistical significance was determined using a one-way ANOVA, followed by a Dunnett's (A) and Tukey's (D) multiple-comparison test [$^{ns}p > 0.1234$ with a 95% confidence level]. Data were plotted and analyzed using GraphPad Prism v9.5.1, KaleidaGraph v4.03, and ImageJ v1.54 (NIH, Bethesda, USA), as applicable, and figures were prepared and assembled using Adobe Illustrator 2023 v27.0.

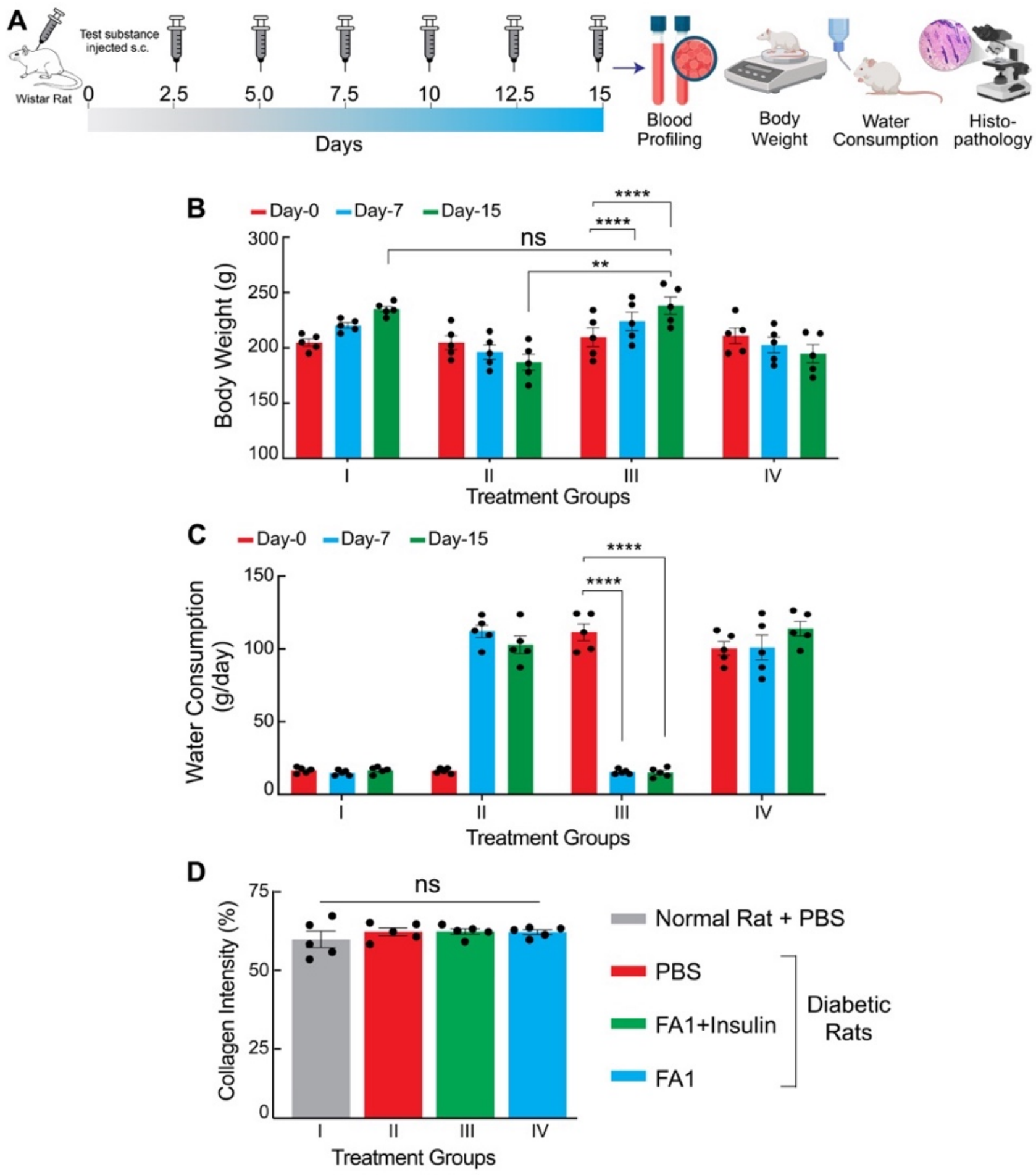

FIGURE S10 | Long-term biosafety assessment of the FA1 amyloid hydrogel. (A) Schematic representation of the *in vivo* biosafety study in which animals received repeated subcutaneous injections of FA1 hydrogel every 2.5 days over a 15-day period. The first group comprised healthy rats receiving subcutaneous PBS, while the second, third, and fourth groups consisted of STZ-induced diabetic rats treated with PBS, insulin-loaded FA1 amyloid hydrogel (FA1+insulin), and FA1 amyloid hydrogel alone, respectively. Some graphical icons in the schematic were created using BioRender.com. (B) Longitudinal body weight change profiles of the rat groups during the 15-day biosafety evaluation across the four treatment groups, showing no significant differences in body weight among the groups following repeated administration. This suggests that the FA1 amyloid hydrogel did not adversely affect body weight. (C) Water consumption profiles of the four experimental groups throughout the 15-day study, demonstrating no treatment-associated alterations. (D) Quantification of collagen deposition intensities at the skin injection sites in four different treatment groups based on Mason's trichrome staining, showing no significant differences among the treatment groups. This implies that the FA1 amyloid hydrogel does not induce collagen deposition and is thus biosafe. For B, C, and D, the data represent the mean ± S.E.M. (n=5), and the statistical significance was determined using a two-tailed paired t-test (B and C) or one-way ANOVA, followed by a Dunnett's multiple-comparison test (D) [$^{****}p < 0.0001$, $^{**}p < 0.0021$, and $^{ns}p > 0.1234$ with a 95% confidence level]. Data were plotted and analyzed using GraphPad Prism v9.5.1, and figures were prepared and assembled using Adobe Illustrator 2023 v27.0.

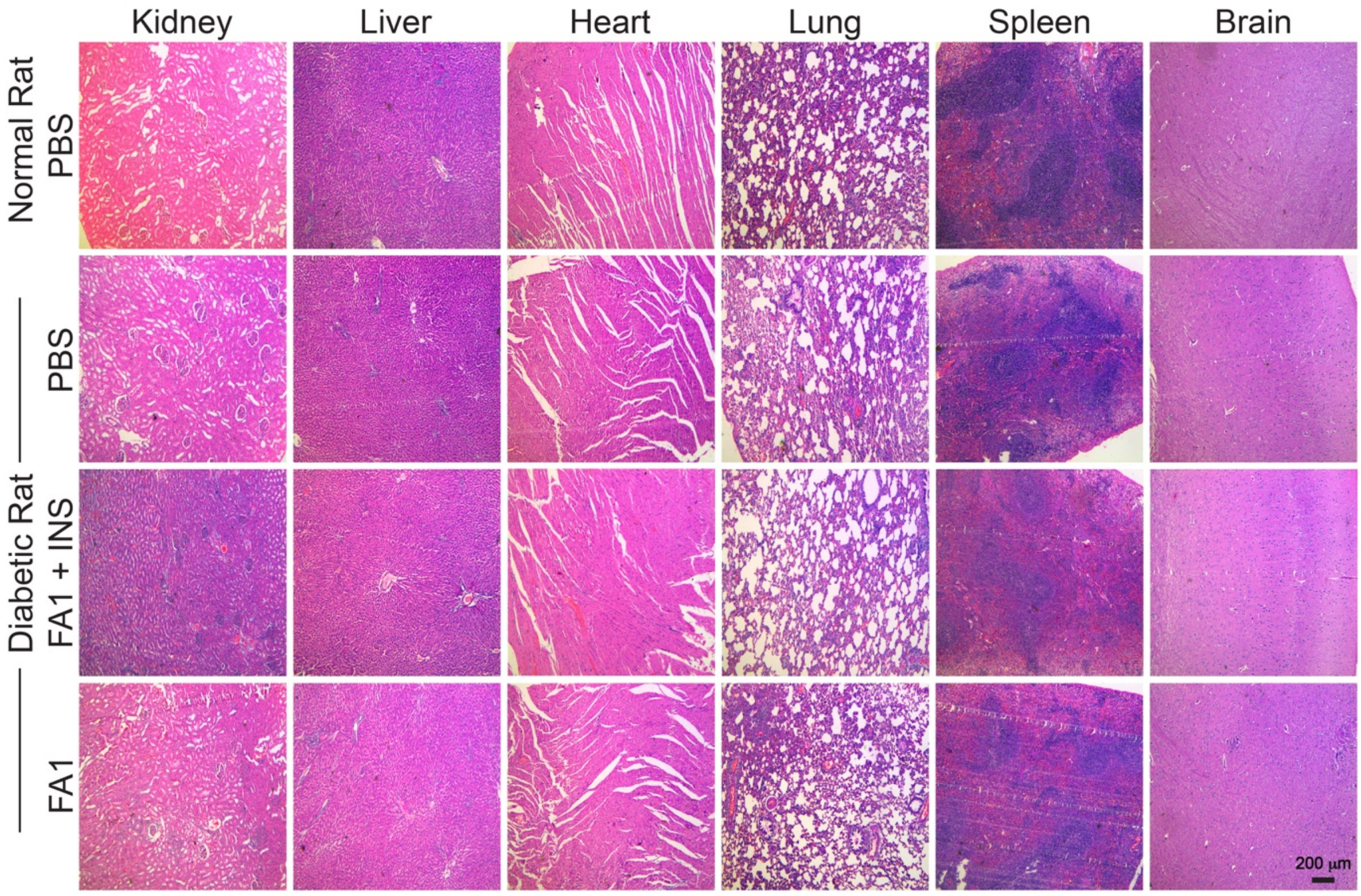

FIGURE S11 | Representative hematoxylin and eosin (H/E)-stained images of tissues from major organs. The stained sections of the major organs were collected following repeated subcutaneous administration of the FA1 amyloid hydrogel every 2.5 days over a 15-day period. Histological examination showed preserved tissue architecture with no observable pathological abnormalities, inflammation, necrosis, or treatment-associated tissue damage compared with the control groups. This implies that the amyloid hydrogel formulation is biosafe to be used for drug delivery applications. Data were analyzed using ImageJ v1.54 (NIH, Bethesda, USA), and the figure was prepared and assembled using Adobe Illustrator 2023 v27.0.

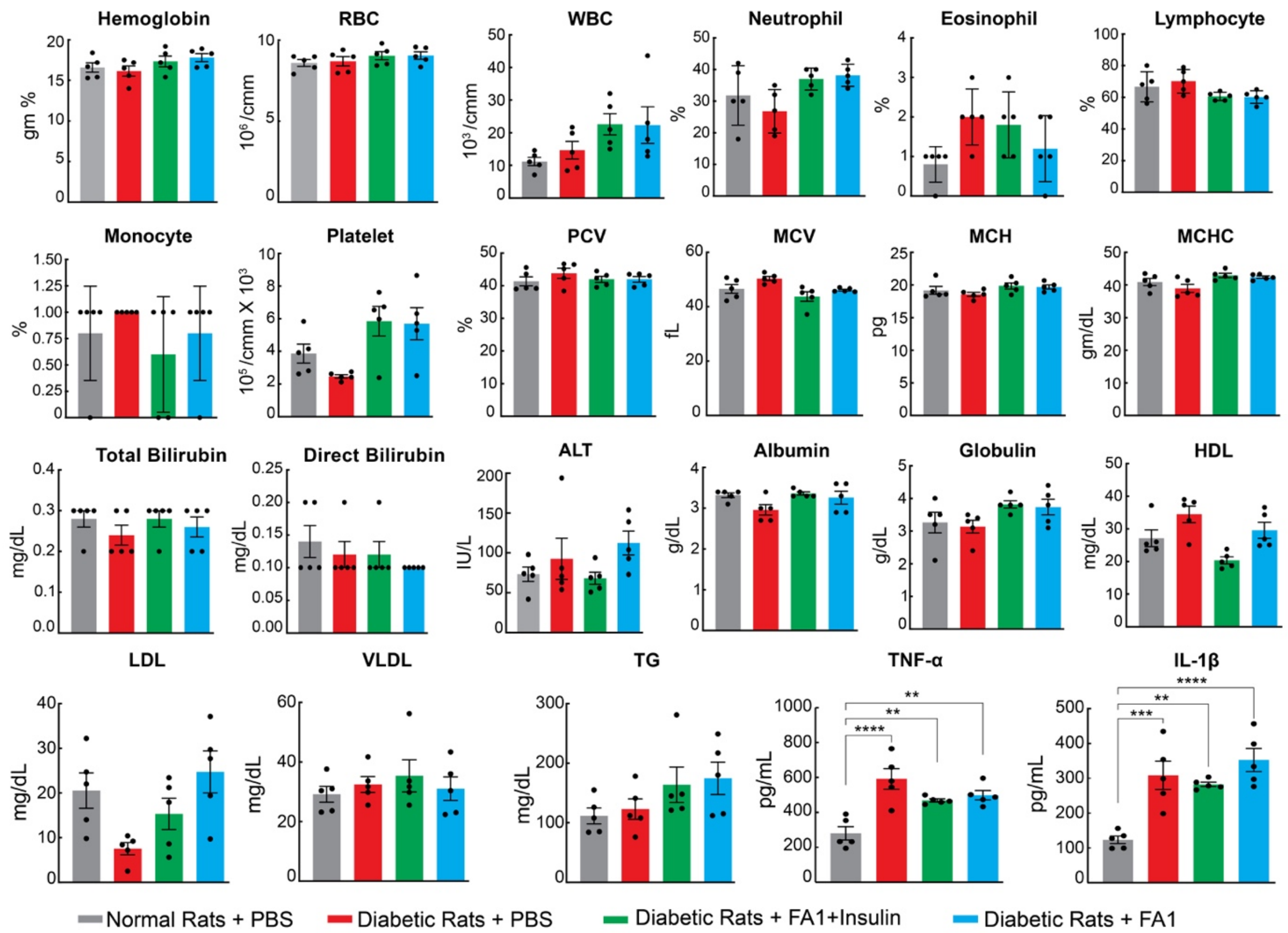

FIGURE S12 | Pre-clinical biosafety evaluation by serum biochemistry and hematological analyses following repeated subcutaneous administration of the FA1 amyloid hydrogel every 2.5 days for 15 days. Serum biochemical markers, including total protein, albumin, globulin, bilirubin, alanine aminotransferase (ALT), and lipid profiles (HDL, LDL, VLDL, total cholesterol, and triglycerides), together with complete hematological indices and pro-inflammatory cytokines (TNF-α and IL-1β), were quantified to evaluate systemic toxicity. No significant alterations were observed relative to the control groups, indicating preserved hepatic, renal, metabolic, and hematological function following repeated administration of the FA amyloid hydrogel. Although a modest increase in TNF-α and IL-1β levels was detected, the response remained within a mild physiological range and was consistent with a transient inflammatory reaction following subcutaneous biomaterial implantation, without evidence of systemic toxicity. Collectively, these findings demonstrate the favorable systemic biosafety profile of the FA1 amyloid hydrogel for drug-delivery applications. The data represent the mean ± S.E.M. (n=5), and the statistical significance was determined using a one-way ANOVA, followed by a Dunnett's multiple-comparison test [****$p < 0.0001$, ***$p < 0.0002$, **$p < 0.0021$ and $^{ns}p > 0.1234$ with a 95% confidence level]. Data were plotted and analyzed using GraphPad Prism v9.5.1, and figures were prepared and assembled using Adobe Illustrator 2023 v27.0.

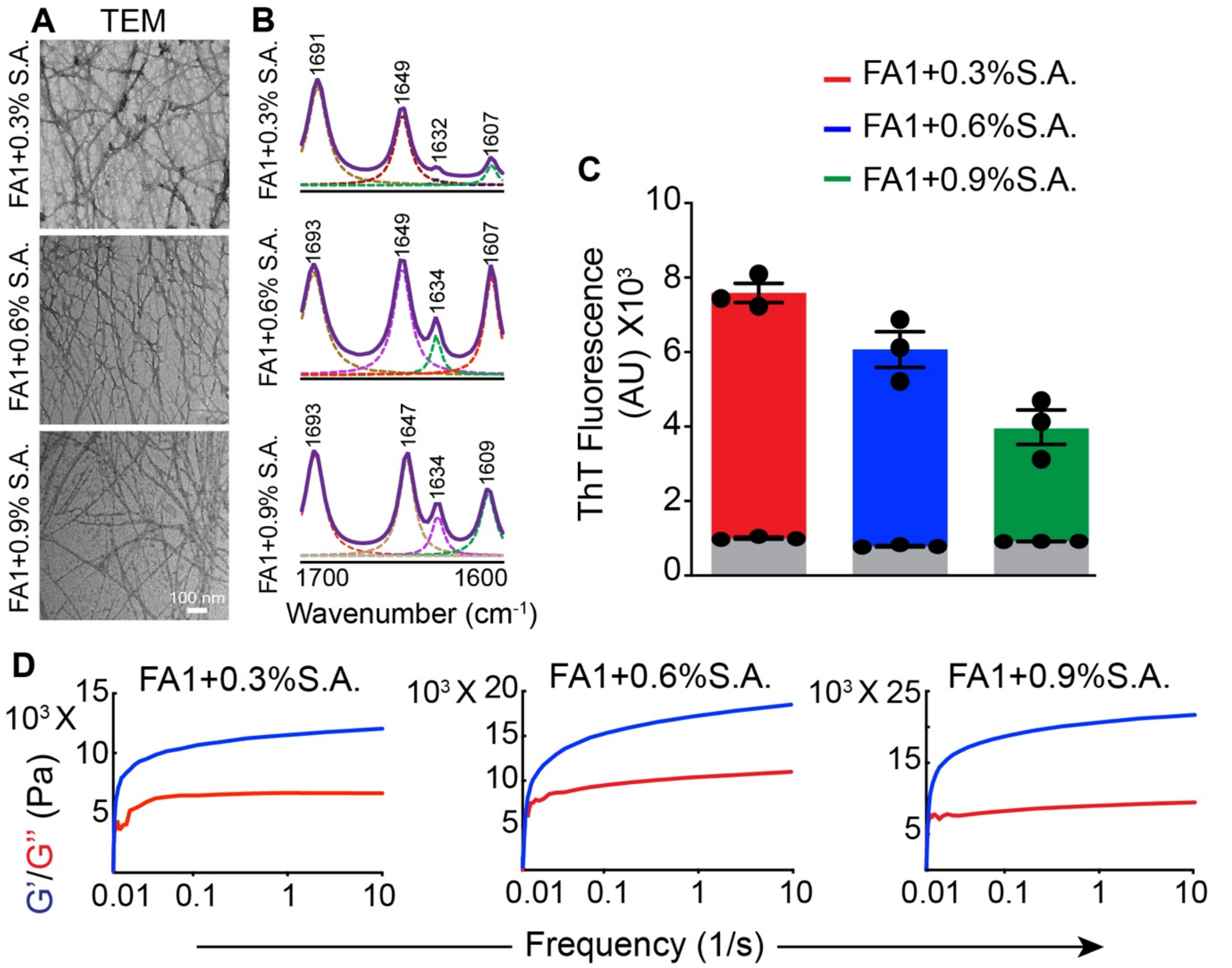

FIGURE S13 | Biophysical characterization of the FA1-sodium alginate (S.A.) composite hydrogels. (A) Transmission electron micrographs of FA1 composite hydrogels containing 0.3%, 0.6%, and 0.9% S.A. All formulations exhibited dense networks of long, unbranched amyloid fibrils with characteristic morphology, demonstrating that incorporation of S.A. did not disrupt amyloid fibril formation. The scale bar for TEM is 100 nm. (B) FTIR spectra of these composite hydrogels (with three different concentrations of S.A.) exhibiting the amide-I region's peaks at about 1630 and 1690 cm^{-1}, implying retention of cross-β-sheet structures after the incorporation of S.A. (C) ThT fluorescence assay of the FA1 amyloid composite hydrogels formulated with 0.3%, 0.6%, and 0.9% S.A., where the existence of amyloidogenic fibrils in the hydrogels is shown at 480 nm following hydrogel binding. The binding of ThT to the respective monomeric pentapeptides is shown by the grey bar inside each individual bar. This again implies that the composite hydrogels retain the characteristic amyloidogenic nature after S.A. incorporation. (D) Frequency sweep rheological test, showing the graph of the composite hydrogel's frequency versus storage modulus (G') and loss modulus (G'') at 37 °C. All formulations exhibited G' values greater than G'' across the tested frequency range, confirming the viscoelastic nature of the composite hydrogels. Data were plotted and analyzed using GraphPad Prism v9.5.1 and ImageJ v1.54 (NIH, Bethesda, USA), as applicable, and figures were prepared and assembled using Adobe Illustrator 2023 v27.0.

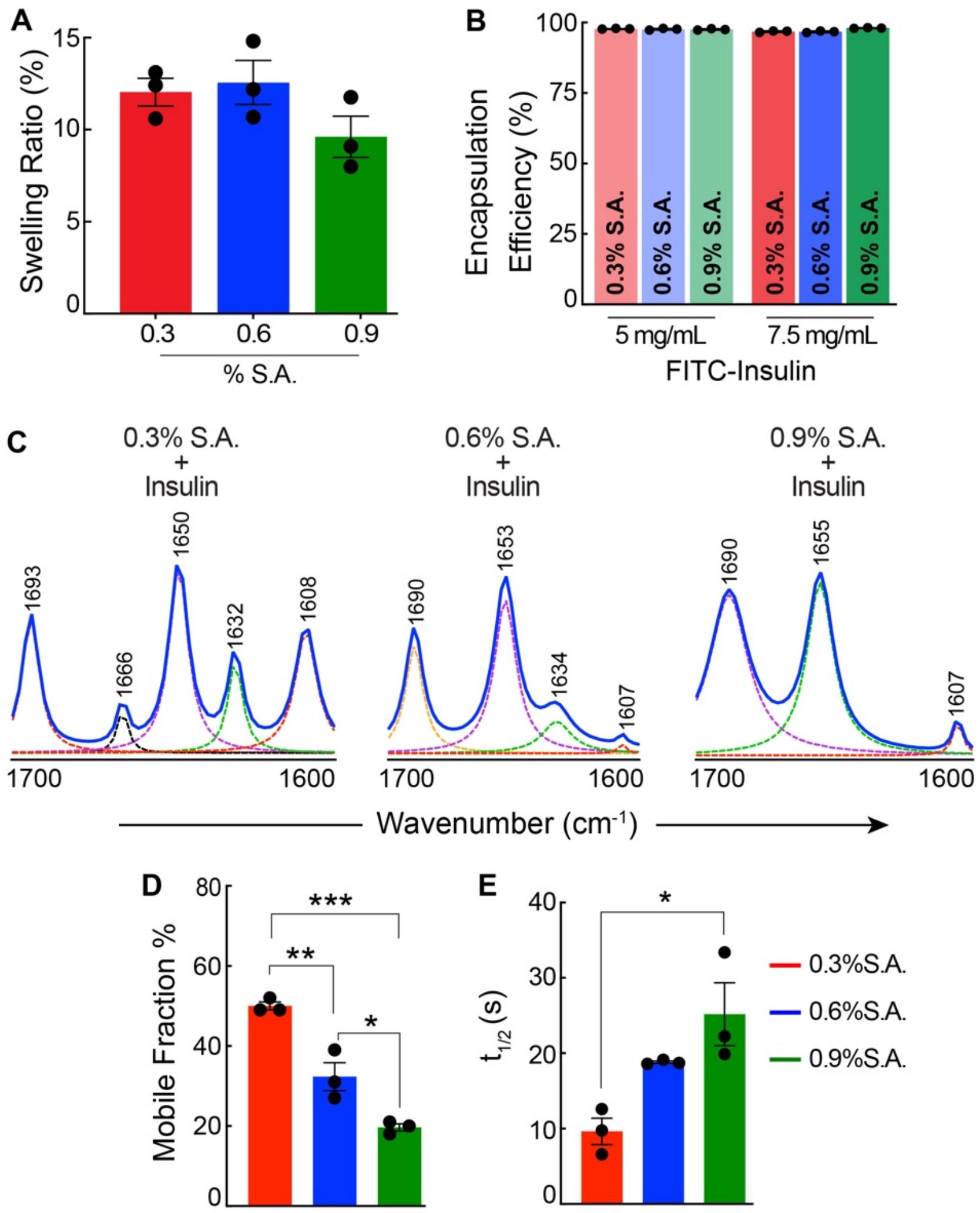

FIGURE S14 | Functional evaluation of the FA1-S.A. composite hydrogel. (A) Swelling percentages of the FA1 composite hydrogels containing 0.3%, 0.6%, and 0.9% S.A. after 24 h, suggesting their non-swelling behavior. (B) Encapsulation efficiency of these composite hydrogels (FA1 with three different concentrations of S.A.) in loading FITC-insulin at 5 and 7.5 mg/mL concentrations. (C) FTIR spectra of insulin-encapsulated composite hydrogels, revealing the presence of an alpha helix (~1650-1655 cm^{-1}), confirming successful encapsulation of insulin and preservation of its native protein conformation within the hydrogel matrix. (D and E) Quantitative analysis of the FRAP experiments showing the % Mobile fraction (D) and calculated $t_{1/2}$ (E) of the FITC-insulin within the composite hydrogels, indicating that both parameters vary as a function of S.A. concentration. For D and E, the data represent the mean ± S.E.M. (n=3), and the statistical significance was determined using a one-way ANOVA, followed by a Tukey's multiple-comparison test [***$p < 0.0002$, **$p < 0.0021$, and *$p < 0.0332$ with a 95% confidence interval]. For simplicity, the composite hydrogel formulations FA1 + 0.3% S.A., FA1 + 0.6% S.A., and FA1 + 0.9% S.A. are denoted as 0.3% S.A., 0.6% S.A., and 0.9% S.A., respectively, throughout the figure. Data were plotted and analyzed using GraphPad Prism v9.5.1 and ImageJ v1.54 (NIH, Bethesda, USA), as applicable, and figures were prepared and assembled using Adobe Illustrator 2023 v27.0.

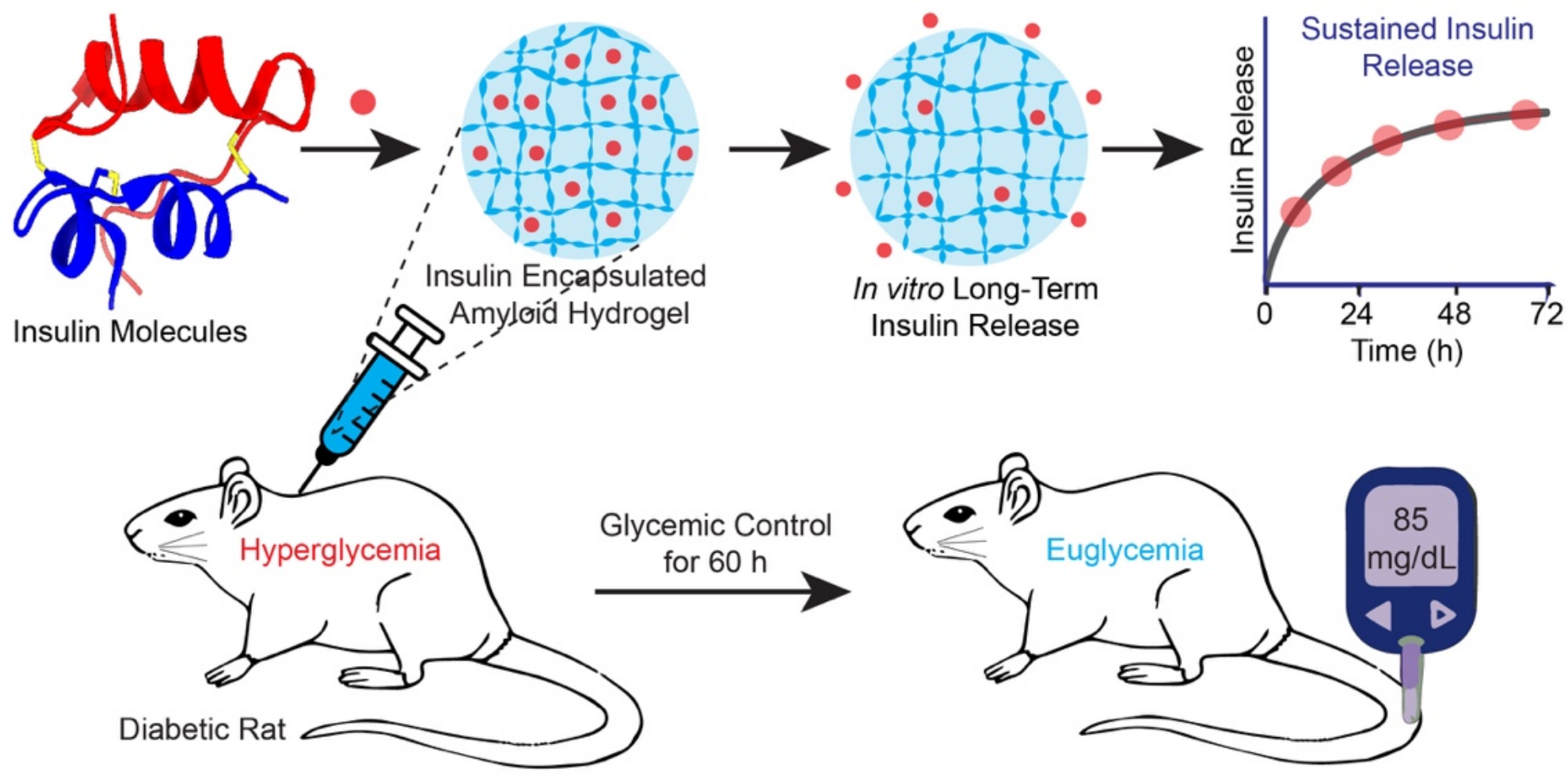

FIGURE S15 | Schematic illustration of sustained insulin delivery from peptide-based functional amyloid hydrogels. Insulin molecules are encapsulated within the hydrogel matrix, which serves as an injectable subcutaneous depot for controlled, sustained insulin release. The amyloid hydrogel enables sustained insulin release for approximately 72 h under *in vitro* conditions, while preserving insulin structural integrity and biological activity. Following a single subcutaneous administration in STZ-induced Type 1 diabetic rats, the formulation maintained normoglycemia for approximately 60 h, demonstrating potent therapeutic efficacy. The insulin structure shown in the schematic was generated from the PDB entry (PDB ID: 3I40), and the figure was prepared and assembled using Adobe Illustrator 2023 v27.0.

Supporting Tables

Table S1 | Coefficient of determination (R^2) values for kinetic modeling of insulin release from two peptide-based functional amyloid hydrogels. Experimental *in vitro* release profiles were fitted to the zero-order, first-order, Higuchi, and Korsmeyer-Peppas models to evaluate the release mechanism of insulin from the hydrogel matrices.[1,2] R^2 was used to evaluate the goodness-of-fit of each kinetic model, with higher R^2 values indicating a better fit of the experimental data to the corresponding kinetic model:

S. No.	Name of the model	Coefficient of determination (R^2)	
		**FA1**	**FA2**
1.	Zero-Order	0.9569	0.9568
2.	First-Order	0.9014	0.8972
3.	Higuchi	0.8088	0.8113
4.	Korsmeyer-Peppas	0.9350	0.9379

Table S2 | Coefficient of determination (R^2) values for kinetic modeling of insulin release from FA1-S.A. composite hydrogels containing different S.A. concentrations. Experimental *in vitro* release profiles from composite hydrogels formulated with 0.3%, 0.6%, and 0.9% S.A. were fitted to the zero-order, first-order, Higuchi, and Korsmeyer-Peppas models to examine the predominant release mechanism of insulin from the composite hydrogel matrices.[1,2] R^2 was used to evaluate the goodness-of-fit of each kinetic model, with higher R^2 values indicating greater agreement between the experimental release profiles and the corresponding kinetic model:

S. No.	Name of the model	Coefficient of determination (R^2)		
		**FA1+0.3% S.A.**	**FA1+0.6% S.A.**	**FA1+0.9% S.A.**
1.	Zero-Order	0.9680	0.9866	0.8847
2.	First-Order	0.9509	0.9610	0.8215
3.	Higuchi	0.9519	0.9491	0.9354
4.	Korsmeyer-Peppas	0.9279	0.8875	0.9169

References

1. T. Higuchi, “Rate of Release of Medicaments from Ointment Bases Containing Drugs in Suspension,” *Journal of Pharmaceutical Sciences 50*, no. 10 (1961): 874–875, https://doi.org/10.1002/jps.2600501018.

2. R. W. Korsmeyer, E. Von Meerwall, and N. A. Peppas, “Solute and Penetrant Diffusion in Swellable Polymers. II. Verification of Theoretical Models,” *Journal of Polymer Science Part B: Polymer Physics 24*, no. 2 (1986): 409–434, https://doi.org/10.1002/polb.1986.090240215.
